# The myopia susceptibility locus vasoactive intestinal peptide receptor 2 (*VIPR2*) contains variants with opposite effects

**DOI:** 10.1038/s41598-019-54619-8

**Published:** 2019-12-03

**Authors:** Kim Hung Leung, Shumeng Luo, Regina Kwarteng, Sin-Guang Chen, Maurice K. H. Yap, Chien-Ling Huang, Shea Ping Yip

**Affiliations:** 1 Department of Health Technology and Informatics, The Hong Kong Polytechnic University, Hong Kong SAR, China; 2 School of Optometry, The Hong Kong Polytechnic University, Hong Kong SAR, China

**Keywords:** Genetic association study, Genetic markers, Genotype, Haplotypes

## Abstract

Myopia is the commonest eye disorder in the world. High myopes are predisposed to ocular pathologies. The vasoactive intestinal peptide receptor 2 (*VIPR2*) gene was identified as a myopia susceptibility locus by our group and another group. We continued to fine-map this locus. A case-control study was performed in 4 sequential stages with a total of 941 highly myopic subjects and 846 control subjects, all unrelated Chinese. Stage 1 experimentally genotyped 64.4% of the entire cohort for 152 single-nucleotide polymorphisms (SNPs) and Stage 2 the remaining subjects for 21 SNPs. Stage 3 combined the genotypes for 21 SNPs for the entire cohort, and identified one group of high-risk haplotypes and one group of protective haplotypes significantly associated with high myopia. Stage 4 imputed genotypes for variants in the *VIPR2* region and identified two independent groups of variants: one group with high-risk minor alleles and another with protective minor alleles. Variants within each group were generally in strong linkage disequilibrium among themselves while high-risk variants were in linkage *equilibrium* with protective variants. Therefore, the *VIPR2* locus seems to contain variants with opposite effects. This is the first study that has examined the genetic architecture of a myopia susceptibility locus in detail.

## Introduction

Refractive error is an ocular disorder whereby the image of an object is not accurately focused on the photoreceptor layer of the retina. Of all types of refractive errors, the commonest is myopia whereby the image of a distant object is focused in front of the retinal photoreceptors in an unaccommodated eye. To a large extent, myopia is caused by an enlarged eyeball that is axially elongated, particularly that of the vitreous chamber^1,2^. Although myopes can still see distant objects clearly by wearing appropriate spectacles of negative lens, the enlarged eyeball remains elongated. The more clinically significant problem caused by myopia is the increased risks of ocular pathologies such as cataract, glaucoma, myopic macular degeneration and retinal detachment, particularly in high myopia^3,4^; and high myopia is usually defined as a refractive error (RE) of -6 diopters (D) or worse^3^. Indeed, the prevalence of visual impairment (including blindness) increases with increasing severity of myopia and older age^5,6^. With reference to emmetropes (those without myopia), the lifetime risk of visual impairment is 3.4-fold higher for high myopes with RE between -6 D and -10 D, and 22-fold higher for extreme myopes with RE of -10 D or worse^5^. This obviously imposes high healthcare cost and high economic burden on the affected populations. The prevalence of myopia has reached or is approaching epidemic levels in some parts of the world, particularly in urbanised cities^5^. In general, myopia is much more prevalent in Oriental populations than in Caucasian populations (80–90% vs 30–50%)^7–10^.

Myopia has been the focus of intense research in recent decades. It is generally accepted that both environmental and genetic factors contribute to myopia development^1,2^. For effective treatment and prevention of myopia, it is crucial to understand the underlying disease mechanisms and biological pathways leading to the development of myopia^1,11,12^. Therefore, it is essential to identify the myopia susceptibility genes and study how different causal variants influence the gene functions and the genetic networks involved. Linkage studies have identified many myopia loci (*MYP1* to *MYP26*)^11–13^. Early linkage analyses mapped many myopia loci (*MYP1* to *MYP22*) to broad chromosomal regions. In contrast, recent linkage analyses, often empowered by whole-exome sequencing, can pinpoint coding mutations (missense, nonsense or frameshift) in specific single genes (*ZNF644*, *CCDC111*, *LRPAP1*, *SLC39A5*, *P4HA2* and *ARR3* representing *MYP21* to *MYP26*, respectively) in multi-generation families with family members affected by high myopia. These rare examples are singe-gene disorders^1^. The other more common type of high myopia usually affects unrelated individuals and, as a type of complex disease^1^, represents one extreme in the entire spectrum of refractive error, which by itself is a complex trait. Many susceptibility variants (and loci) for refractive error have robustly been found by meta-analyses of genome-wide association studies (GWAS) carried out, for example, by the Consortium for Refractive Error and Myopia (CREAM), 23andMe or both^11–15^.

Vasoactive intestinal peptide (VIP) has been shown to be involved in different model animals (chicks, mice and monkeys) in the development of refractive properties in normal eye and in myopic eye induced by form deprivation^16–20^. In particular, a positive correlation was demonstrated between the *VIP* expression and the vitreous chamber depth^20^. Vasoactive intestinal peptide receptor 2 (VIPR2) is one of the two known VIP receptors and the chromosomal location of the *VIPR2* gene is on 7q36, which is within a putative locus for autosomal dominant high myopia (previously known as *MYP4*)^21,22^. In myopia induced in chicks by form deprivation, the *VIPR2* expression in the retina and the choroid was up-regulated in the treated eyes with reference to the fellow control eyes^23^, and the induced myopia could be suppressed by a non-selective antagonist of VIP receptors in a dose-dependent fashion^24^. All these suggested that *VIPR2* was a good positional and functional candidate gene for myopia susceptibility. With this background, we used a candidate-gene approach and identified the *VIPR2* gene to be highly associated with high myopia in the Han Chinese population^25^. In particular, the strongest association signal (omnibus test, *P* = 9.10 × 10^−10^) came from a haplotype window consisting of four tag single-nucleotide polymorphisms (SNPs), namely rs2071623, rs2071625, rs2730220 and rs885863. Independently and simultaneously, another group used a hypothesis-free approach and conducted meta-analysis of GWAS, and also found a single SNP (rs2730260; overall *P* = 8.98 × 10^−14^) within the *VIPR2* gene highly associated with high myopia in Han Chinese^26^. Both case-control studies tested myopia as a *qualitative* trait (high myopia) and investigated tag SNPs that were selected with the linkage disequilibrium (LD) measure r^2^ ≥ 0.8 from SNPs genotyped in the International HapMap Project^27^. Therefore, the associated SNPs were more likely tagging other un-genotyped causal genetic variants driving the genetic association. *VIPR2* is expressed in many different human tissues including the retina and the retinal pigment epithelium^26,28–30^. With great excitement while halfway through our current investigation, a mega-study further established *VIPR2* as a susceptibility locus for the *quantitative* trait refractive errors (and age of diagnosis of myopia) in an extremely large cohort of participants of mainly European origin^15^.

Although over 200 genetic variants have been identified by genetic association studies, particularly GWAS, to influence myopia susceptibility^11–15^, the genuine causal variants, their functions and the underlying mechanisms how these variants are linked to the development of myopia remain to be investigated. As a logical step forward in elucidating the genetic network involving *VIPR2* in the medium term and the etiology of myopia in the long run, we set out to fine-map this region with a view to identifying putative causal variants. This study reports this follow-up work. To our surprise, we find that, within the *VIPR2* gene and its immediate flanking regions, there are two independent groups of genetic variants with opposite effects as measured by odds ratio (OR).

## Methods

### Study subjects

The study recruited 1,787 unrelated Chinese individuals with age ranging from 18 to 50 years through the Optometry Clinic of The Hong Hong Polytechnic University as we have reported previously^31–33^. The inclusion criteria for cases and controls were based on the refractive error in terms of spherical equivalent (SE), which is calculated as the sum of sphere diopters and half-cylinder diopters. A refraction of SE of at least -8 D for both eyes defined a case subject while an SE within ± 1 D for both eyes defined a control subject. We excluded from the study any individual with ocular disorder (e.g. cataract and glaucoma) or inherited disorder (e.g. Stickler syndrome and Marfan syndrome) that has an association with myopia. We obtained approval from the Human Subjects Ethics Subcommittee of The Hong Hong Polytechnic University, and adhered to the tenets of the Declaration of Helsinki. We also obtained written informed consent from all subjects. Eye examination was performed as described previously^31–33^. Briefly, every subject received a complete ophthalmic examination, which included refraction, visual acuity and dilated examination of the fundus in the Optometry Clinic of the University. In particular, an open-field autorefractor (SRW-5000; Shin-Nikkon) was used to measure objective refraction after 1% tropicamide (2 drops per eye) was instilled in the eye of the subject. A-mode ultrasonography (Advent A/B System, Mentor) was used to measure axial length after 0.4% benoxinate hydrochloride (1 drop per eye) was used to anaesthetize the eye.

The study was carried out in 4 stages. Stage 1 included 1,151 subjects (691 cases and 460 cases) with genotypes for 152 SNPs (see results). Stage 2 involved 636 subjects (250 cases and 386 controls) with genotypes for 21 SNPs. Stage 3 included all 1,787 subjects and combined the data of the first two stages for 21 SNPs. Stage 4 further examined imputed genotype data for 368 high-quality SNPs (info score > 0.3) for the entire sample set.

### Selection and genotyping of SNPs

For this study, we identified 196 proxy SNPs that were tagged by the associated SNPs (rs2071623, rs2071625, rs2730220, rs885863 and rs2730260)^25,26^ at r^2^ ≥ 0.4 based on the pilot Asian data of the 1000 Genomes Project (Phase 1) by SNAP Proxy Search^34^. We further added 25 more proxy SNPs that were tagged by the associated SNPs with r^2^ values of 0.2–0.4, but had an annotation score of 3a or less by RegulomeDB^35^. RegulomeDB is an online tool annotating the functional features of noncoding variants of the human genome, and a score of 3a or less means that the SNP of interest has at least three known or predicted regulatory features. Upon removing duplicate SNPs, there were 202 SNPs.

These 202 SNPs were genotyped by the custom-made Infinium iSelect BeadChips (Illumina), which also included ~9,000 other SNPs for other studies. Of these 202 SNPs, 4 were removed by us because of low final assay design score (<0.4) generated by the Assay Design Tools (Illumina) during assay design stage, and 29 more by the vendor (Illumina) because of manufacturing failure. Finally, 169 SNPs were included in the tailor-made iSelect BeadChips for this study. The genotyping was performed as a contract service by the Centre for Genomic Science (University of Hong Kong) according to the manufacturer’s instructions. Two important SNPs removed by the vendor from the BeadChips were later genotyped by an in-house method called unlabelled probe melting analysis^25^ (see supplementary methods). These two SNPs were rs3812302 (with a RegulomeDB annotation score of 3a) and rs2071625 (one of the associated SNPs).

We used the GenomeStudio software (version 2.0, Illumina) to call genotypes based on the colour (green or red) and intensity of the fluorescence signals according to the vendor’s instructions. We adjusted the cut-off value of the GenCall (a measure of genotyping accuracy) to 0.05, and samples with scores below the threshold were excluded. We also filtered out low-quality samples with a low GenTrain score (<0.7497), which is the cluster algorithm used in GenomeStudio and represents the quality of SNP calling.

After analysis of Stage-1 genotype data, we followed up 21 SNPs in the Stage-2 study with 636 case-control samples. Of these, 20 SNPs were genotyped using either the MassARRAY iPLEX assays (Agena) or the unlabelled probe melting analysis (see supplementary methods), and one SNP (rs114961653; also coded as vr106 in this article) failed to be genotyped by either method.

### Imputation of genotypes

Although the sense strand of *VIPR2* is on the minus-strand sequence of chromosome 7, we coded the genotypes of all SNPs based on the plus strand before imputation to ensure consistency with the genotype data of the reference panels of the 1000 Genomes database. We used IMPUTE2 to impute (a) sporadic missing genotypes of genotyped SNPs, and (b) the genotypes of un-genotyped SNPs from ~57.5 kb downstream of *VIPR2* (rs262134; also coded as vr001 in this paper) to 20 kb upstream of *VIPR2*. IMPUTE2 was provided with all available reference haplotypes, and designed in such a way that it would choose a custom reference panel for each individual to be imputed^36^.

For Stage-1 study, the input data for imputation were the genotypes of 152 SNPs for 1,151 subjects. For Stage-2 study, the input data were the genotypes of 20 SNPs for 636 subjects; the genotypes of rs114961653 (vr106) were entirely imputed. After imputation, the imputed SNPs were filtered out if any one of the following conditions occurred: (1) imputation info score <0.3, (2) minor allele frequency (MAF) < 1%, or (3) *P* < 0.001 for the Hardy-Weinberg equilibrium (HWE) test of the genotypes of control subjects (see below). We used the genotype dosage data for association analysis.

### Statistical analysis

For Stages 1 to 3, we compared cases and controls for sex ratios by chi-squared test, mean age by unpaired t test, and genotypes by the software package PLINK (version 1.07)^37^. We tested the genotypes of control subjects for HWE by means of exact test with *P* = 0.001 as the significant threshold^38^. Logistic regression was used to test for association between the phenotype high myopia and the SNPs (single markers or haplotypes) based on additive model with adjustment for sex and age as covariates; the significance level was indicated as *P*_*a*_. ORs were calculated accordingly.

For haplotype analysis, we adopted a sliding-window strategy^37^ with window size varying from 1 to 15 SNPs per window; a maximum of 15 SNPs per window (~10% of 152 SNPs for Stage-1 study) was chosen to strike a balance between exhaustiveness and penalty imposed by multiple testing. For a given window size, all possible windows of the same size were tested with a shift of one SNP at a time towards the 3′ end of the gene. Two sets of tests were performed for haplotype association. One was an omnibus test (invoked by the commands --hap-window and --omnibus), which jointly assessed the haplotypic effects of each sliding window as a single test of (H − 1) degrees of freedom, where H is the number of haplotypes with frequency of at least 0.01 for the window under study. Another one was a general test (invoked by the command --hap-window) of every haplotype with a frequency of at least 0.01 within the window concerned, and OR was calculated for this particular haplotype with reference to the remaining haplotypes within the same window; this implies that the reference haplotypes are different for different haplotypes.

Multiple testing was corrected by permutation testing to give empirical *P* values based on at least 10,000 permutations across all SNPs, all haplotypes or all haplotype windows. *P* value was indicated as *P*_*aemp*_ if adjusted for sex and age, and corrected for multiple comparisons with *P*_*aemp*_ < 0.05 indicating significant association.

For Stage 4 based on genotype imputation, we compared the genotype dosages of cases and controls for association with high myopia by the software package SNPTEST (version 2.5.2)^39^ under additive model. Multiple testing was corrected by means of Benjamin-Hochberg procedure^40^ at a false discovery rate of 0.05 with the corresponding *P*_*cor*_ < 0.05 indicating significant association. ORs were calculated accordingly. Regional Manhattan plot was created using the R package qqman^41^. To plot LD patterns for SNPs examined at Stage 4, the imputation genotype probability score results were transformed to the genotypes with threshold probability score of at least 0.8, and the LD patterns constructed by Haploview^42^.

## Results

### Characteristics of study subjects

As shown in Table 1, there were significantly fewer males in the case groups than in the respective control groups as assessed by chi-squared test: 31.3% vs 42.2% for Stage 1 (*P* = 2.761 × 10^−4^), 28.0% vs 41.5% for Stage 2 (*P* = 5.213 × 10^−4^), and 30.4% vs 41.8% for Stage 3 (*P* = 5.278 × 10^−7^). Note that Stage-3 study included all 1,787 subjects from the first two stages. When comparing the mean age by unpaired t test, we found that the case subjects were older than the control subjects for Stage 1 (31.6 vs 30.4 years; *P* = 0.0291) while the case subjects were younger than the control subjects for Stage 2 (28.7 vs 34.8 years; *P* = 3.883 × 10^−11^) and for Stage 3 (30.8 vs 32.4 years; *P* = 0.0019). In view of these results, we always adjusted for sex and age in subsequent comparisons of genotypes between cases and controls.

**Table 1:** Characteristics of study subjects.

	Stage-1 subjects (N = 1,151)		Stage-2 subjects (N = 636)		Stage-3 subjects (N = 1,787)	
	Cases	Controls	Cases	Controls	Cases	Controls
Total number	691	460	250	386	941	846
Proportion of males, %	31.3	42.2	28.0	41.5	30.4	41.8
Age (mean ± SD), years	31.6 ± 8.8	30.4 ± 9.5	28.7 ± 9.1	34.8 ± 13.7	30.8 ± 9.0	32.4 ± 11.8
SE (mean ± SD), D	−10.37 ± 2.46	0.07 ± 0.52	−10.19 ± 2.51	0.06 ± 0.48	−10.32 ± 2.47	0.07 ± 0.51
AL (mean ± SD), mm	27.66 ± 1.21	23.73 ± 0.82	27.48 ± 1.23	23.72 ± 0.81	27.61 ± 1.22	23.73 ± 0.81

Stage-3 study combined all the subjects from both Stage-1 and Stage-2 studies and hence the total number of subjects is the sum of the first two stages (1,787 = 1,151 + 636). The ocular measurements (SE and AL) are based on the data of the right eyes. SD: standard deviation; SE: spherical equivalent; D: diopter; AL: axial length.

Table 1 shows the ocular measurements only for the right eyes because the measurements were highly correlated between the right and the left eyes: r = 0.9658, 0.9618 and 0.9655 for SE, and r = 0.9624, 0.9625 and 0.9636 for axial length (AL) for Stage 1, Stage 2 and Stage 3 respectively. The mean SE ranged from −10.19 to −10.37 D for cases, and from 0.06 to 0.07 D for the controls across the 3 stages of study. The mean AL ranged from 27.48 to 27.66 mm for cases, and from 23.72 to 23.73 mm for the controls across the 3 stages of study. The criteria for subject recruitment dictated the skewed distribution of these 2 ocular measurements.

### Stage-1 to Stage-3 studies

Details and main findings of the 4 stages of the study are summarised in Table 2. Details of the results for Stage-1 to Stage-3 studies are described in Supplementary results. We summarise these results concisely in the following paragraphs.

**Table 2:** Summary of details and main findings in 4 different stages of the *VIPR2* study.

	Stage 1	Stage 2	Stage 3	Stage 4
No. of SNPs analysed	152	21	21	368
Methods of genotyping	iSelect BeadChips; 2 SNPs by UPMA. Experimentally determined	iPLEX assays & UPMA. Experimentally determined	iSelect BeadChips, IPLEX assays & UPMA. Experimentally determined	Imputation by IMPUTE2
No. of subjects studied	1,151	636	1,787	1,787
Single-marker analysis	No significant SNP	1 significant SNP	17 significant SNPs	197 significant SNPs divided into 2 groups (24 being high-risk and 173 being protective)
Haplotype analysis	50 significant SWs 1–14 SNPs per SW 21 SNPs involved	19 significant SWs 2–11 SNPs per SW 21 SNPs involved	204 significant SWs 310 significant haplotypes divided into 2 categories (high-risk or protective)	—
Details shown in	Suppl. Fig. 1 Suppl. Table 1 Suppl. Table 2	Suppl. Table 3 Suppl. Table 4	Suppl. Table 5 Suppl. Table 6	Suppl. Table 7 Fig. 1 Suppl. Fig. 2 Suppl. Fig. 3 Fig. 2

SNP: single-nucleotide polymorphism; UPMA: Unlabelled probe melting analysis; SW: Sliding Window; Suppl.: Supplementary.

For *Stage-1 study*, we analysed the genotypes of 152 SNPs and coded them as vr001 to vr152 (Supplementary Fig. 1) in the sequential order of chromosomal positions on the plus strand. Single-marker analysis did not show any association with high myopia upon correction for multiple testing (Supplementary Table 1). For sliding-window haplotype analysis with window size varying from 1 to 15 SNPs per window, there were 2,175 sliding windows. Of these, only 50 sliding windows remained significantly associated with high myopia after multiple-testing correction (*P*_*aemp*_ < 0.05, Supplementary Table 2). These significant windows varied in size from 2 to 14 SNPs per window and involved 21 SNPs from vr107 (rs73523914) to vr087 (rs2730224), both inclusive. Therefore, we followed up these 21 SNPs in Stage 2.

For *Stage-2 study*, only one SNP (vr099 or rs73169220) were marginally significant (*P*_*aemp*_ = 0.0492; Supplementary Table 3). For sliding-window haplotype analysis involving 210 sliding windows, only 19 sliding windows were significantly associated with high myopia after multiple-testing correction (Supplementary Table 4).

For *Stage-3 study*, we combined the genotype data of 21 SNPs (from vr107 to vr087) from the first two stages and hence the total sample size was 1,787 subjects. Single-marker analysis showed that 17 SNPs were significantly associated with high myopia (Supplementary Table 5). The minor alleles of these associated SNPs were all protective in nature with the OR below 1.0 (ranging from 0.6551 to 0.7438, Supplementary Table 5). With sliding-window haplotype analysis (Supplementary Table 6), 204 out of 210 *sliding windows* showed significant association with high myopia, and 310 out of 778 *haplotypes* of varying window sizes with frequency > 0.01 were significantly associated with high myopia. Upon careful examination, significant haplotypes could be divided into two distinct categories: high-risk haplotypes or protective haplotypes (Supplementary Table 6). Of the 310 significant haplotypes with different window sizes, 195 (62.9%) were *high-risk* in nature with OR ranging between 1.32 and 2.30. On the other hand, 115 (37.1%) out of 310 significant haplotypes were *protective* in nature with OR between 0.74 and 0.65. Protective haplotypes had a window size varying from 1 to 14 SNPs and were all formed by minor alleles of the constituent SNPs. In fact, 16 SNPs each alone had a protective minor allele/haplotype (Supplementary Table 6), and hence were putative causal variants on their own – a finding consistent with single-marker analysis.

### Stage-4 study

**Figure 1:**
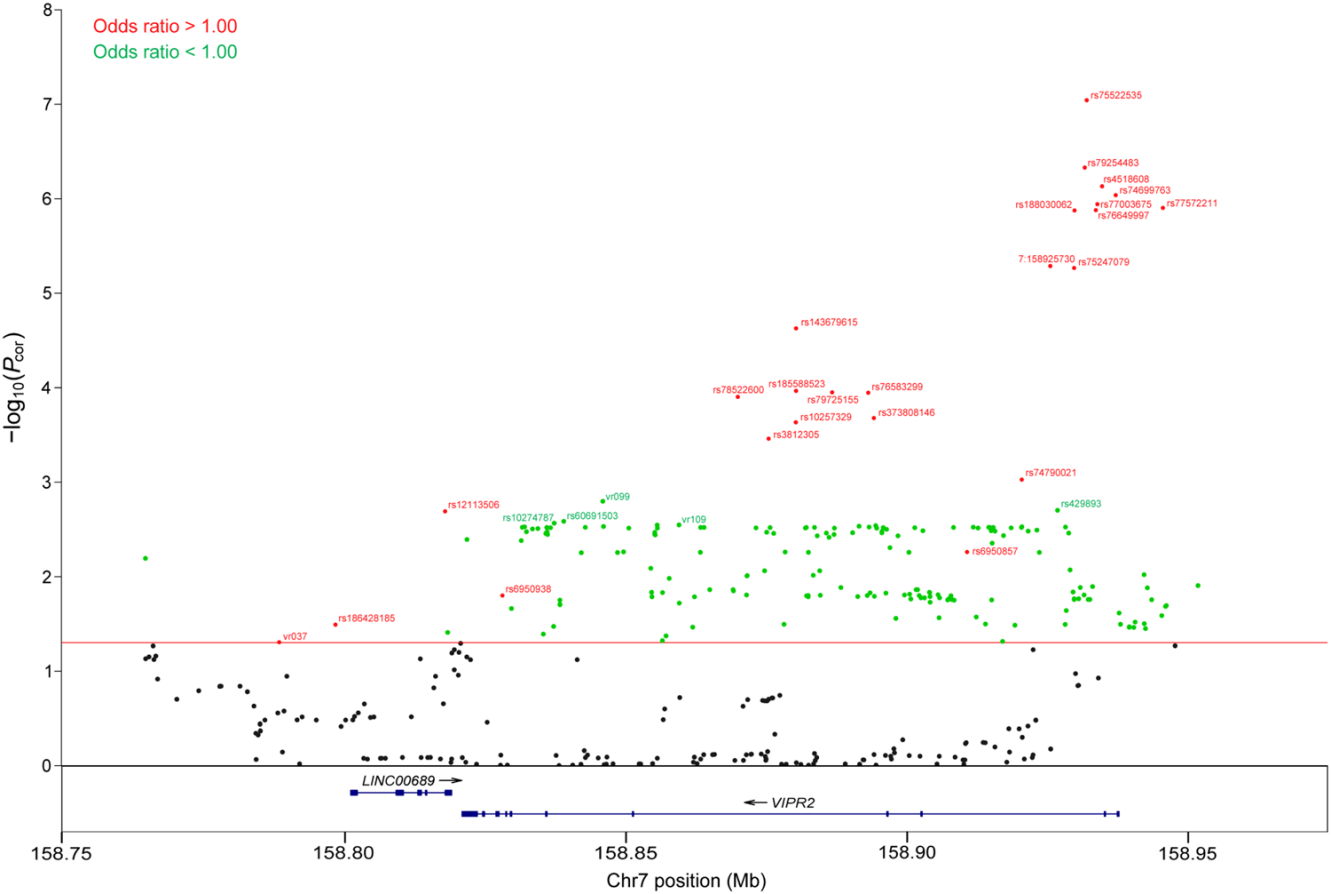
Regional Manhattan plot for the *VIPR2* region for Stage-4 study. In total, 368 variants were compared for high myopia in case-control subjects by SNPTEST based on genotype dosage under additive model. Multiple testing was corrected by means of Benjamini-Hochberg procedure at a false discovery rate of 0.05 with the corresponding *P*_*cor*_ < 0.05 (indicated by the red line in the figure) indicating significant association. Only variants above the red line are identified. High-risk variants (odds ratio, OR > 1.00) are shown in red, and protective variants (OR < 1.00) in green for easy tracking. The identities of protective variants are only shown for the top 5 to avoid cluttering of the figure.

After imputation and filtering, 368 SNPs and indels were available for single-marker analysis. After multiple-testing correction based on a false discovery rate of 0.05, 197 SNPs/indels remained significantly associated with the phenotype high myopia (*P*_*cor*_ < 0.05, Fig. 1 and Supplementary Table 7). The least significant SNP was vr037 (rs76749764) (*P* = 0.026; *P*_*cor*_ = 0.049). Consistent with the results of Stage-3 study, the significant SNPs/indels could also be divided into two distinct groups: variants with high-risk minor alleles or variants with protective minor alleles (hereafter called high-risk SNPs and protective SNPs respectively, and shown in red and green respectively in Fig. 1).

Out of 197 significant variants (SNPs/indels), 24 had high-risk minor alleles with OR ranging between 1.21 and 2.60 (Supplementary Table 7). Of these 24 high-risk variants, 20 were in moderate (r^2^ of 0.40–0.70) to strong (r^2^ > 0.70) LD with each other (Supplementary Fig. 2). The remaining 4 variants were in weak LD (r^2^ < 0.40) with the other 20 variants although vr037 (rs76749764) and rs186428185 (no. 26 and 34 among the 368 SNPs under study; Supplementary Table 7) formed one strong-LD pair with each other. The rows showing these 4 variants in Supplementary Table 7 are highlighted in grey for easy tracking. Among the 20 variants mentioned above, the OR ranged from 1.35 to 1.80 and the MAFs were in the range of 0.0633–0.1310 in case subjects and 0.0366–0.0913 in control subjects with the MAFs always higher in cases than in controls (Supplementary Table 7).

Out of the 197 significant variants, 173 had protective minor alleles with OR between 0.84 and 0.48 (Supplementary Table 7). Of these 173 protective variants, 165 were in moderate to strong (r^2^ ≥ 0.4) LD among themselves while the remaining 8 variants were in very weak LD with these 165 variants (Supplementary Fig. 7). If we focused on the 165 variants in moderate to strong LD among themselves, the OR ranged between 0.84 and 0.67, and the MAFs were in the range of 0.0690–0.1868 in cases and 0.0871–0.2293 in controls with MAFs always lower in cases than in control. Of the 173 protective variants, 111 were newly identified by the imputation approach and had never been genotyped in Stage-1 study.

**Figure 2:**
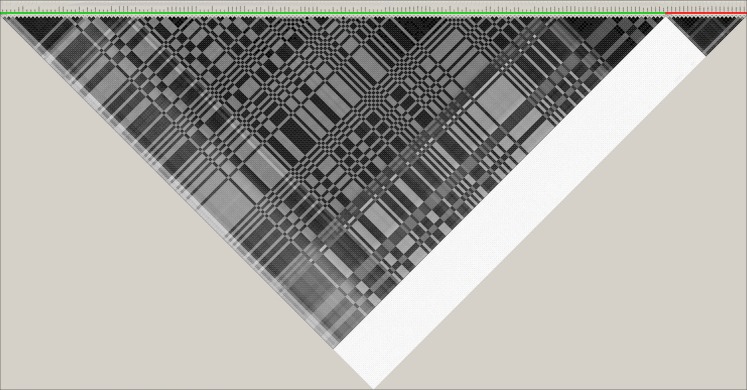
The linkage disequilibrium (LD) pattern of high-risk and protective variants identified in Stage-4 study. The LD measures are shown as r^2^ values for cases and controls together with black indicating r^2^ = 1, white indicating r^2^ = 0, and shades of grey indicting 0 < r^2^ < 1. Without affecting the interpretation, some variants were purposely left out here to help visualise the independence (i.e. very weak LD) between these two groups of variants. High-risk variants are underlined in red and shown on the right according to chromosomal positions within their own group while protective variants are underlined in green and shown on the left according to chromosomal positions within their own group.

Figure 2 shows the LD relationship (r^2^) among the high-risk variants and the protective variants; without affecting the interpretation, some variants were deliberately left out in Fig. 2 to help visualise the independence (very weak LD) between these groups of variants. Strikingly, high-risk variants were in almost linkage *equilibrium* (r^2^ < 0.05) with protective variants and vice versa even though they were located within the *VIPR2* gene within a genomic region of ~200 kb and interspersed among each other.

## Discussion

This study was carried out in 4 sequential stages. Single-marker analysis did not show promising results with the first 2 stages (Supplementary Tables 1 and 3). Sliding-window haplotype analysis identified 21 SNPs for follow-up in Stage 2 (Supplementary Table 2), and also both high-risk and protective haplotypes in Stage 3 (Supplementary Table 6). In Stage-4 study, single-marker analysis confirmed the findings of Stage 3, and identified one group of high-risk variants and another group of protective variants (Fig. 1 and Supplementary Table 7). Analysis of the LD structure in the study subjects illuminated their independence of one another. First, the variants within each group were generally in strong LD with each other with a few exceptions (Supplementary Figs. 2 and 3). Second, these 2 groups of variants (high-risk vs protective) were in linkage *equilibrium* with each other (Fig. 2) even though they were located within the same gene in a small genomic region (~200 kb). This means that the high-risk allele and the protective allele are carried on two different haplotype backgrounds at the *VIPR2* locus and will not usually be found in the same individuals except in a small proportion of individuals who happen to carry these two different haplotypes by random chance.

The study was carried out in 4 stages (see Methods). The first 3 stages could have been combined into a single stage by genotyping all 152 SNPs for all 1,787 samples. However, the latter approach is obviously much more expensive than the first 3 stages of the current study, which only genotyped 21 SNPs for all 1,787 samples with the remaining SNPs being genotyped in 1,151 samples only. Indeed, a stepwise design with a joint analysis (Stage 3 in our study) of the first 2 stages has been shown to be more efficient and cost-effective^43^. We made best use of the 3 first stages of our study through a sliding-window-based haplotype analysis, which enabled a much smaller number of SNPs (21 out of the original 152 in Stage 1) to be genotyped in Stage 2, and also empowered the discovery of 2 distinct groups of haplotypes (high-risk vs protective; Supplementary Table 7) in Stage 3. This latter finding is consistent with the presence of 2 distinct groups of variants (high-risk vs protective) discovered in Stage 4. The relatively high density of SNPs genotyped in the first 2 stages enabled imputation of genotypes for un-genotyped SNPs in Stage 4. Certainly, imputation of genotypes based on available reference genome sequences is now a common strategy for fine-mapping purposes because it makes available the genotypes of more variants only at the expense of more computational time and also increases the power of genetic association studies^44^.

We used iSelect BeadChips for genotyping SNPs in Stage 1. The cost structure of iSelect BeadChips dictated that the minimum number of samples for a given SNP content was 1,152. This in turn dictated the number of subjects for Stage-1 study, and hence a smaller size for Stage-2 study for a given total sample size for the entire study. In the end, our Stage-1 study tested 1,151 distinct samples together one duplicate sample as an internal quality (QC) check. The iSelect BeadChip is a well-established high-throughput commercial genotyping platform, which incorporates many quality control checks to ensure reliable genotype calls (see Methods). Our internal quality check included one sample genotyped in duplicate and the genotypes were 100% matched between the duplicates (see Supplementary results). MassARRAY iPLEX assay is also a reliable commercial genotyping platform (Agena Biosciences) for medium throughput, which is well suited for our Stage-2 study. Similarly, genotype calls based on the iPLEX assay also incorporate many QC checks to ensure accuracy. Both platforms are based on the principle of single-base extension although their signal readouts are different^45,46^. For both systems, no genotype will be called if the QC checks fail. On the other hand, unlabelled probe melting analysis is an in-house low-throughput genotyping method^25^ used in the first 2 stages for a few SNPs, and is based on the method reported by Zhou *et al*.^47^. Direct DNA sequencing of representative samples was used to confirm all observed genotypes^25^. In other words, all 3 genotyping methods are well-established and reliable with adequate QC checks to ensure accuracy of genotype calls. They were used in the current study for different levels of throughput.

**Figure 3:**
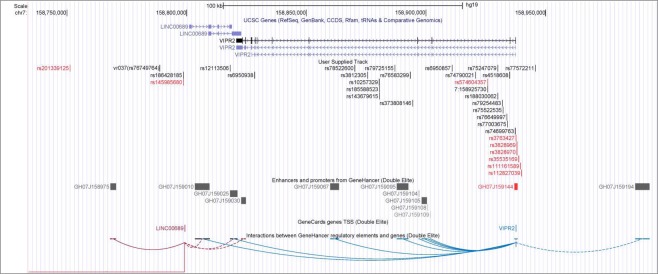
The locations of high-risk variants, promoter and enhancers and the gene-enhancer interactions in the *VIPR2* region. High-risk variants (indicated in black) and other variants in strong LD with them (r^2^ ≥ 0.7; indicated in red) are shown together with regulatory genomic elements (promoter and enhancers). Gene-enhancer interactions are indicated by arcs linking relevant regulatory elements. Note that some variants are located in the promoter/enhancer GH07J159144 and in the enhancer GH07J159025, and both regulatory elements interact with each other.

Intriguingly, one high-risk SNP (rs74699763) and 3 other SNPs (rs3763427, rs3828969 and rs3828370) in strong LD with the high-risk SNP group are located at the promoter/enhancer (GH07J159144, derived from 3 information sources by GeneHancer^48^) of the *VIPR2* gene (Fig. 3). This promoter/enhancer element can be bound by 22 different transcription factors, 20 of which are expressed in the retina^29^. In addition, another high-risk SNP (rs12113506) is located within another enhancer element (GH07J159025, derived from 2 information sources by GeneHancer^48^), which interacts with the promoter/enhancer GH07J159144 of the *VIPR2* gene as documented by GeneHancer^48^. This bioinformatics analysis provides clues to the putative functional effects of some high-risk SNPs on *VIPR2* expression.

The recent mega-study of refractive errors reported 2 lead SNPs in the *VIPR2* region: rs60884546 located within a *VIPR2* intron, and rs7789096 located ~140 kb upstream of *VIPR2* transcription start site. Our study imputed genotypes for SNPs within 20 kb upstream of *VIPR2* and therefore did not look into rs7789096; even if we tried, the imputation accuracy would be expected to be very low because of the linkage equilibrium between this SNP and the SNPs we experimentally genotyped in this study. On the other hand, rs60884546 was indeed imputed, but later removed because its MAF was 0.0074 (lower than the threshold MAF of 0.01). This is not unexpected because rs60884546 has an MAF of 0.005 for Chinese (CHB) in the 1000 Genomes Database. By contrast, its MAF is 0.025 as documented in the recent study of refractive errors^15^ and for Europeans (CEU) in the 1000 Genomes Database. In addition, its minor allele has an effect of reducing the diopters of refractive errors (i.e. more myopic) and hence is equivalent to a high-risk allele for the qualitative trait of myopia.

It is intriguing that our 24 high-risk SNPs (Fig. 1 and Supplementary Table 4) are in almost complete linkage *equilibrium* with rs60884546 (Supplementary Fig. 5). This suggests that there are 2 distinct genuine high-risk causal variants in the *VIPR2* locus. For our high-risk SNPs, the MAFs are in the range of 0.011–0.152 (median = 0.070) for our control subjects (a selected group because myopia is very common in the Hong Kong Chinese population), 0.005–0.170 (median = 0.087) for Chinese (CHB) in the 1000 Genomes Database, and 0.000–0.111 (median = 0.010) for Europeans (CEU) in the 1000 Genomes Database. In brief, the MAFs of these high-risk SNPs are higher in Chinese than in Europeans – a scenario opposite to that for rs60884546. It is tempting to speculate that population histories and perhaps natural selection might have a role to play in the differential MAFs of these 2 groups of high-risk SNPs in these 2 ethnic groups. It is also appealing to hypothesise that the much higher MAFs for our high-risk SNPs in Chinese than for the high-risk rs60884546 in Europeans may contribute to the much higher prevalence of myopia in Chinese than in Europeans.

Of our 173 protective SNPs, 124 (72%) are predicted expression quantitative trait loci (eQTLs) based on a recent publicly available database of retinal transcriptome^30^; here, a cis-eQTL was defined by a SNP being within a genomic distance of 1 Mb either upstream or downstream of the transcriptional start site of a gene. This lends support to our finding^49,50^ and helps us prioritise these SNPs (and other SNPs in strong LD with them) for follow-up functional investigation. Just like high-risk variants, some protective variants and other SNPs in strong LD with this protective SNP group are located in regulatory genomic elements (promoter and/or enhancers, Supplementary Fig. 4). This analysis also provides hints on the putative functional effects of some protective variants on *VIPR2* expression.

## Conclusion

This study first used experimentally determined genotypes and sliding-window haplotype analysis to identify two groups of susceptibility haplotypes in the *VIPR2* locus for high myopia in a Chinese population, namely one group of high-risk haplotypes and one group of protective haplotypes. Subsequent genotype imputation and single-marker analysis identified two independent groups of *VIPR2* variants associated with the qualitative phenotype high myopia: one group of high-risk SNPs and another group of protective variants. To the best of our knowledge, this is the first study that has examined the genetic architecture of a confirmed myopia susceptibility locus in detail.

## Supplementary information

Supplementary Information

## Data Availability

The summary statistics dataset of the current study is freely available from the corresponding authors upon reasonable requests.
