# Supplementary material for: The myopia susceptibility locus vasoactive intestinal peptide receptor 2 (VIPR2) contains variants with opposite effects
Source: Sci Rep. 2019 Dec 3;9:18165. doi: 10.1038/s41598-019-54619-8 (PMC6890636; doi:10.1038/s41598-019-54619-8)
Supplement: Supplementary file 1 — Supplementary Information [file 41598_2019_54619_MOESM1_ESM.pdf]

The myopia susceptibility locus vasoactive intestinal peptide receptor 2 (*VIPR2*) contains variants with opposite effects

Kim Hung Leung[1@], Shumeng Luo[1@], Regina Kwarteng[1], Sin-Guang Chen[1], Maurice K.H. Yap[2], Chien-Ling Huang[1*], Shea Ping Yip[1*]

[1]Department of Health Technology and Informatics, The Hong Kong Polytechnic University, Hong Kong SAR, China. [2]School of Optometry, The Hong Kong Polytechnic University, Hong Kong SAR, China.

[@]Kim Hung Leung and Shumeng Luo contributed equally.

*Correspondence and requests for materials should be addressed to S.P.Y. (email: shea.ping.yip@polyu.edu.hk) or C.L.H. (email: cl.huang@polyu.edu.hk).

Supplementary information

Table of contents (Supplementary information)

Item	Supplementary materials	Page
1.	**Supplementary methods**	3
2.	**Supplementary results**	6
3.	**Supplementary Table 1.** Stage-1 study: Summary of *VIPR2* genotype data and single-marker association testing	8
4.	**Supplementary Table 2.** Stage-1 study: Summary of significant omnibus tests for sliding-window haplotype analysis of 152 VIPR2 single-nucleotide polymorphisms (SNPs)	12
5.	**Supplementary Table 3.** Stage-2 study: Summary of *VIPR2* genotype data and single-marker association testing	13
6.	**Supplementary Table 4.** Stage-2 study: Summary of significant omnibus tests for sliding-window haplotype analysis of 21 VIPR2 single-nucleotide polymorphisms (SNPs)	14
7.	**Supplementary Table 5.** Stage-3 study: summary of VIPR2 genotype data and single-marker association testing	15
8.	**Supplementary Table 6.** Summary of sliding-window haplotype analysis of the entire sample (Stage 3): Omnibus tests and significant haplotypes	16
9.	**Supplementary Table 7.** Summary of single-marker analysis of imputed genotypes: significant SNPs	21
10.	**Supplementary Figure 1**. The locations of single-nucleotide polymorphisms (SNPs) tested in Stage 1 as arranged along the plus strand of chromosome 9.	24
11.	**Supplementary Figure 2.** The linkage disequilibrium (LD) pattern of high-risk variants identified in Stage-4 study.	25
12.	**Supplementary Figure 3.** The linkage disequilibrium (LD) pattern of protective variants identified in Stage-4 study.	26
13.	**Supplementary Figure 4.** The locations of protective variants, promoter and enhancers and the gene-enhancer interactions in the *VIPR2* region.	27
14.	**Supplementary Figure 5.** The linkage disequilibrium (LD) pattern of high-risk variants identified in Stage-4 study and the “high-risk” variants (rs60884546 and rs7789096) identified in a recent mega-study of refractive errors.	28

Supplementary Methods

Unlabelled probe melting analysis (UPMA). The following table shows the sequences of primers for polymerase chain reaction (PCR) and the sequences of unlabelled probes for melting analysis.

SNP (genotyped by UPMA)	Sequences (5'>3') of primers & probes (F: Forward primer; R: Reverse primer; P: unlabelled probe)* [Lim: Limiting primer; Exc: Excess primer]	PCR		
		product size (bp)	Tm (°C)	Protocol
**Stage 1**				
rs3812302 (vr102)	F: CGTCCTGAGAAGCCTGGT [Lim]<br>R: TTTTCTCCCGTCTCCGTCG [Exc]<br>P: GCGGAGCCCCATCCCCAGGGCCCTGCAGtttt	185	58	C3
rs2071625 (vr082)	F: CCAACAAGAAAAACCACTGTTTC [Lim]<br>R: TGTAGGTTTGGGGTCACTCA [Exc]<br>P: AATTCCCTCACAAGGACACACCTGTGTaaaa	190	58	C2
**Stage 2**				
rs7787641 (vr105)	F: CAGTGCTGACCCAAAGAACAA [Exc]<br>R: CACCAATCAGTACCCTGTCAAAA [Lim]<br>P: AGCCTGTAGAGACAACTCACTGGGTCaaaa	249	58	C2
rs2540345 (vr104)	F: GCAGTGCTGACCCAAAGAAC [Lim]<br>R: ATGGACCAATCAGTAGGATGAG [Exc]<br>P: CCACAGCACATAAGGAGACCCAGTGAAaaaa	180	55	C2
rs3812302 (vr102)	See above (Stage 1)			
rs73169220 (vr099)	F: CAAAACAAATGGAAGGATCTACTATGA [Lim]<br>R: TTCTCTCAAAAAAACAAAAAAAACAAAAATTCTATT [Exc]<br>P: CTATAGATACCATGCAATACCAAATGAtttt	149	55	C1
rs2730264 (vr097)	F: CAAAACAAATGGAAGGATCTACTATGA [Lim]<br>R: TTCTCTCAAAAAAACAAAAAAAACAAAAATTCTATT [Exc]<br>P: ATTCAGATGCTAGTGTTTTAAGACATGtttt	160	55	C2
rs56236179 (vr096)	F: GGTGACAGAGACATACTCCA [Lim]<br>R: ATTCTCCATTTCTTTATCTGTTTTCTTA [Exc]<br>P: AATAGCAATTCAGATGCTAGTGTTTTAtttt	160	55	C2
rs73169218 (vr094)	F: ACACAGAGCCAGCCTACAAA [Lim]<br>R: CTTGGTCTTTATTGGTCGCTG [Exc]<br>P: GGAAATCTCTATCAAACAACTAGCAGAtttt	158	58	C2
rs2540359 (vr092)	AAAGGAGGTCAGTGAAAATGGTG [Lim]<br>CCAGGACCAAGTACCCACAA [R]<br>CTTAGCCGGGGGAAGGTCCTGAATAaaaa	227	58	C2
rs2730254 (vr091)	F: AGGAGGTCAGTGAAAATGGTG [Lim]<br>R: GTGGAAAAGGGTGAGCTAAAGA [Exc]<br>P: ACAACAAAGATACTCAAAGGCTTATGCtttt	157	58	C2
rs886656 (vr088)	F: CAGCGGCAAAAGAGAAAAATAAAGT [Lim]<br>R: CACTCTTCCTGCGTCCTCT [Exc]<br>P: CCACATGAGGGGCCGTGTACCGCCTtttt	169	58	C2

* A 3'-tail of 4 bases (aaaa or tttt) is always attached to the unlabelled probes to provide 4-base mismatches with the strand of the amplicon complementary to the probe and hence prevents extension of the probe during melting analysis. An Underlined base represents an internal mismatch that is purposely introduced in the unlabelled probe to increase the difference in the melting temperatures between the 2 alleles of the SNP concerned.

Asymmetric PCR was carried out in a 15-µl reaction mixture containing 40 ng DNA templates, 1.5 mM $MgCl_2$, 0.2 mM of each dNTP, 0.1 µM limiting primer, 0.5 µM excess primer (see the Table above), 1× PCR buffer, and 1 U of HotStar Taq Plus DNA Polymerase (Qiagen). There were 2 exceptions. For rs2071625 (vr082), the reaction mixture contained 0.3 µM limiting primer and 1.5 µM excess primer. For rs3812302 (vr102), the reaction mixture also contained 1× Q solution provided by the manufacturer (Qiagen). Amplification was carried out in a Veriti 96-well Thermal Cycler (Applied Biosystems), and comprised 1 cycle of initial denaturation (10 min/95°C), 55 cycles of a 3-step protocol, and a final extension step (7 min/72°C). There were 3 types of the 3-step protocol: C1 (5s/95°C, 5s/Tm and 5s/72°C), C2 (15s/95°C, 15s/Tm and 15s/72°C), and C3 (40s/95°C, 40s/Tm and 40s/72°C) (see the Table above).

After PCR, a 10-µl mixture was prepared for melting analysis: 2 µM Syto 9 (Invitrogen), 0.5 µM unlabelled probe, and 9.1 µl of PCR product. Melting analysis was performed with the "Tm calling" analysis mode on a LightCycler 480 PCR System (Roche Applied Systems), and the melting data were collected with 5 acquisitions/°C while the temperature was increased at a ramping rate of 0.11°C/s from 40°C to 95°C.

MassARRAY iPLEX assay. The iPLEX assay measures the masses of single-base-extended primers on a mass spectrometer to call the genotypes of SNPs. All the reagents and instruments used for this assay were from Agena Biosciences. In Stage 2, 10 SNPs were genotyped by iPLEX assay in 2 multiplex groups (one group of 9 SNPs and another group of 2 SNPs). The following Table shows the sequences of the PCR primers and the primers for single-base extension (SBE). For each multiplex group, PCR was carried out a 5-µl reaction mixture comprising 20 ng DNA templates, 4 mM $MgCl_2$, 0.5 mM of each dNTP, 0.1 µM of each primer, 1× PCR buffer, and 1 U of PCR. Amplification was carried out in a Veriti 96-well Thermal Cycler (Applied Biosystems) with the following thermal cycling condition: 1 cycle of initial denaturation (2 min/95°C), 45 cycles of 30s/95°C, 30s/56°C and 60s/72°C, and a final extension step (5 min/72°C). PCR products were cleaned up in a 7-µl reaction mixture containing 0.5 U of shrimp alkaline phosphatase, 0.24× reaction buffer and 5 µl of PCR product, and the PCR plate was placed in a thermal cycler (40 min/37°C and 5 min/85°C).

SBE was then carried out in 9-µl reaction mixture containing 7 µl of purified PCR products, 0.73 µM and/or 1.46 µM of each extension primer (see the Table below), 0.222× iPLEX Gold Buffer, 1× iPLEX Termination Mix, 1× iPLEX Enzyme. Linear amplification of SBE products was accomplished with the following thermal cycling conditions: an initial denaturation of 30s/94°C; 40 cycles of a 3-step

reaction (5s/94°C, 5s/52°C and 5s/80°C) and a nested 5 cycles of a 2-step reaction (5s/52°C and 5s/80°C) after each 3-step cycle; and a final extension step (3 min/72°C). SBE products were purified with CLEAN Resin, spotted onto a 384-well SpectroChip with MassARRAY Nanodispenser RS1000, and finally analysed on MassARRAY Analyser 4 according to the manufacturers' instructions. Genotypes were called using the MassARRAY Typer 4.0 software after cluster analysis based on default settings. In case of doubt, Sanger sequencing of the amplicons was used to confirm the genotypes. Assays with an overall call rate of less 90% were excluded and therefore the assay for rs3793220 (vr099) was rejected.

**SNP (genotyped by iPLEX assay)**	**Sequences (5'>3') of primers (F: Forward primer; R: Reverse primer; E: Extension primer)**	**PCR product size (bp)**	**Final conc. of extension primer (μM)**
**Group 1**			
rs2730224 (vr087)	F: ACGTTGGATGACAGCTTGTGGAAAGATGCC<br>R: ACGTTGGATGCATTTTGCCTGTCTGGTGGG<br>E: CCTGGAGTGTGCATG	120	0.73
rs73169215 (vr089)	F: ACGTTGGATGTCCCTCTGTCCTCACAGAGC<br>R: ACGTTGGATGTCCAAAGCCCAGGTGCATGT<br>E: ACAGAGCCAGTGCCCC	106	0.73
rs73169216 (vr090)	F: ACGTTGGATGATGTGGGGAGACCCTATTAC<br>R: ACGTTGGATGAACAAGATTTTTCACCTGGC<br>E: TTTTCACCTGGCAAGCCC	107	0.73
rs7784586 (vr093)	F: ACGTTGGATGGGTGGTCATTTTGAAGACAG<br>R: ACGTTGGATGCACAACTCCTTGGGTTTATG<br>E: TGAAGACAGAAGCTCATG	103	0.73
rs2540357 (vr095)	F: ACGTTGGATGTCTGAGATGGCTGTGTGTTG<br>R: ACGTTGGATGATTTCCCCCAAGTACTCGCC<br>E: GCTGAGAAATAAACCAGAAA	100	1.46
rs2730260 (vr100)	F: ACGTTGGATGCACAGGTGGATTATGGACAG<br>R: ACGTTGGATGCTTGAGTCCTCTTAAGAAATC<br>E: GTGGATTATGGACAGAAACAT	119	1.46
rs56375711 (vr103)	F: ACGTTGGATGCGGCAAGTCACAGCCACAG<br>R: ACGTTGGATGAACACCTCGCCAGGACCCC<br>E: CAGCCACAGCAGCCC	103	0.73
rs73523914 (vr107)	F: ACGTTGGATGCTGCTGGGGTGGGAGAGGG<br>R: ACGTTGGATGCCAGTGGCTCCGGGGCAG<br>E: gaATTTCACGGGAGCCC	120	0.73
**Group 2**			
rs2730265 (vr098)	F: ACGTTGGATGGTAGATGAAAAATCCTCAAC<br>R: ACGTTGGATGCATTCCTAAATTAACCCTAC<br>E: AATTAACCCTACATGGTTTT	117	0.73
rs3793217 (vr101)	F: ACGTTGGATGCAATGTCCAGCGACCTTCCT<br>R: ACGTTGGATGGTCAAGTACTTTACAGATG<br>E: cCAGATGTTAACATCATCAGG	120	0.73

Supplementary Results

Stage-1 study. As mentioned in the Methods section, the tailor-made iSelect BeadChips included 169 out of 202 SNPs initially selected for this study. Of these 169 SNPs, 5 were filtered out after quality checks of the raw data, and this gave a success rate of 97.0% for genotyping. Fourteen SNPs were found to be monomorphic for our sample set of 1,151 subjects. One sample was purposely genotyped in duplicate as an internal quality check, and the genotypes were 100% matched between the duplicates. Among the remaining 150 SNPs successfully genotyped with iSelect BeadChips, a call rate of 99.8% was achieved per SNP and per sample. Together with 2 other SNPs genotyped by unlabelled probe melting analysis, we analysed the genotypes of 152 SNPs for Stage-1 study and, for the sake of convenience, coded them as vr001 to vr152 (Supplementary Figure 1) in the sequential order of chromosomal positions on the plus strand.

The genotypes of all 152 SNPs were in Hardy-Weinberg equilibrium (HWE, $P > 0.05$; Supplementary Table 1) for the control subjects and hence all SNPs were tested for association with high myopia. Single-marker analysis did not show any association with high myopia upon correction for multiple testing (Supplementary Table 1). For sliding-window haplotype analysis with window size varying from 1 to 15 SNPs per window, there were 2,175 sliding windows. Of these, only 50 sliding windows remained significantly associated with high myopia after multiple-testing correction ($P_{aemp} < 0.05$, Supplementary Table 2). These significant windows varied in size from 2 to 14 SNPs per window and involved 21 SNPs from vr107 (rs73523914) to vr087 (rs2730224), both inclusive. Therefore, we followed up these 21 SNPs in Stage 2.

Stage-2 study. The genotypes of all 21 SNPs were in HWE ($P > 0.05$; Supplementary Table 3) for the control subjects and hence all SNPs were tested for association with high myopia. After correction for multiple testing, one SNP (vr099 or rs73169220) were marginally significant ($P_{aemp} = 0.0492$). For sliding-window haplotype analysis with window size ranging from 1 to 15 SNPs per window, there were 210 sliding windows. Of these, only 19 sliding windows were significantly associated with high myopia after multiple-testing correction (Supplementary Table 4).

Stage-3 study. Stage 3 combined the genotype data of 21 SNPs (from vr107 to vr087) from the first two stages and hence had a total sample size of 1,787 subjects. The genotypes were all in HWE in the control subjects (Supplementary Table 5). Single-marker analysis showed that 17 SNPs were significantly associated with high myopia with vr099 (rs73169220) giving the lowest P_{aemp} value (0.0003, Supplementary Table 5). The minor alleles of these associated SNPs were all protective in

nature with the OR below 1.0 (ranging from 0.6551 to 0.7438, Supplementary Table 5). With sliding-window haplotype analysis (Supplementary Table 6), 204 out of 210 sliding windows showed significant association with high myopia ($P_{aemp} < 0.05$ with the lowest $P_a = 2.60 \times 10^{-7}$ for the 2-SNP window of vr100-vr099), and 310 out of 778 haplotypes of varying window sizes with frequency > 0.01 were significantly associated with high myopia ($P_{aemp} < 0.05$ with the lowest $P_a = 1.03 \times 10^{-6}$ for the 8-SNP haplotype TTACGTCT of vr106-vr099). Upon careful examination, significant haplotypes could be divided into two distinct categories: high-risk haplotypes or protective haplotypes (Supplementary Table 6).

Of the 310 significant haplotypes with different window sizes, 195 (62.9%) were *high-risk* in nature with OR ranging between 1.32 and 2.30. To form these high-risk haplotypes, vr100 (rs2730260), vr098 (rs2730265) and vr097 (rs2730264) contributed their minor alleles while the remaining constituent SNPs contributed their major alleles (minor and major alleles are shown in red and blue, respectively, in Supplementary Table 6). Of note, 114 high-risk haplotypes had OR between 1.89 and 2.30 (rows highlighted in pink, Supplementary Table 6); their estimated frequencies were 0.0683–0.0788 in cases and 0.0326–0.0438 in controls. Two sub-haplotypes were crucial: the CT sub-haplotype of vr100-vr099, and the TC sub-haplotype of vr097-vr096 (shown in boldface in pink rows, Supplementary Table 6). Their presence, either alone or in combination, would bring the OR up to 1.89 or above. In parallel, high-risk haplotypes encompassing the critical 2-SNP haplotypes, either alone or in combination, tended to give P_a values for association much lower than those for high-risk haplotypes without these sub-haplotypes (10^{-5} – 10^{-6} vs 10^{-3}, Supplementary Table 6).

On the other hand, 115 (37.1%) out of 310 significant haplotypes were *protective* in nature with OR between 0.74 and 0.65. The majority of protective haplotypes (113 out of 115) had OR between 0.70 and 0.65; their estimated frequencies were 0.0831 – 0.1091 in case subjects and 0.1132 – 0.1484 in control subjects (Supplementary Table 6). With one exception (vr099, $P_a = 5.08 \times 10^{-5}$), all protective haplotypes gave P_a values for association in the order of 10^{-3}. Protective haplotypes had a window size varying from 1 to 14 SNPs and were all formed by minor alleles of the constituent SNPs. In fact, 16 SNPs each alone had a protective minor allele/haplotype (Supplementary Table 6), and hence were putative causal variants on their own – a finding consistent with single-marker analysis. (There were a lot more haplotypes involved in haplotype analysis than in single-marker analysis, and this explained why one *less* SNP was significant upon multiple-testing correction in haplotype analysis.) Therefore, in most of such instances, adding more SNPs to form protective haplotypes of larger window size did not change the odds ratios, haplotype frequencies and P_a values too much.

Supplementary Table 1. Stage-1 study: summary of *VIPR2* genotype data and single-marker association testing

	Sequential	Alleles	Genotype counts (11/12/22) (N=1,151)		Minor allele freq.		HWE (Controls)	Association (additive, adjusted for sex & age)	
SNP*	No.*	(1/2)@	Cases (n=691)	Controls (n=460)	Cases	Controls	*P* value	P_a	P_{aemp}
rs56382838	vr152	C/T	5/106/580	5/95/360	0.0839	0.1141	0.8187	0.0209	0.3178
rs9691129	vr151	G/C	6/103/582	3/95/362	0.0832	0.1098	0.3371	0.0370	0.4735
rs3828963	vr150	A/T	5/105/581	5/94/361	0.0832	0.1130	0.8190	0.0222	0.3335
rs67373805	vr149	G/A	5/105/581	5/93/362	0.0832	0.1120	1.0000	0.0266	0.3827
rs59630301	vr148	C/T	5/106/580	5/93/362	0.0839	0.1120	1.0000	0.0262	0.3791
rs56157635	vr147	A/G	5/104/582	5/93/362	0.0825	0.1120	1.0000	0.0226	0.3379
rs17837881	vr146	A/G	5/104/582	5/93/362	0.0825	0.1120	1.0000	0.0226	0.3379
rs56376138	vr145	C/T	5/105/581	5/97/358	0.0832	0.1163	0.8196	0.0110	0.1968
rs17837880	vr144	A/G	5/104/582	5/93/362	0.0825	0.1120	1.0000	0.0226	0.3379
rs76844436	vr143	C/T	139/355/197	81/240/139	0.4580	0.4370	0.2184	0.3234	0.9964
rs73169264	vr142	T/C	5/106/580	5/94/361	0.0839	0.1130	0.8190	0.0211	0.3203
rs3793237	vr141	A/G	5/104/582	5/94/361	0.0825	0.1130	0.8190	0.0189	0.2954
rs399867	vr140	T/C	31/198/462	10/143/307	0.1881	0.1772	0.1999	0.4529	0.9999
rs55776245	vr139	A/G	5/104/582	5/93/362	0.0825	0.1120	1.0000	0.0241	0.3563
rs6459924	vr138	G/A	15/170/506	15/136/309	0.1447	0.1804	1.0000	0.0187	0.2938
rs3793232	vr137	G/A	16/168/507	15/136/309	0.1447	0.1804	1.0000	0.0184	0.2902
rs3793231	vr136	T/C	15/169/507	15/137/308	0.1440	0.1815	1.0000	0.0136	0.2300
rs6459922	vr135	A/G	15/169/507	15/137/308	0.1440	0.1815	1.0000	0.0136	0.2300
rs55708631	vr134	A/G	5/103/583	5/94/361	0.0818	0.1130	0.8190	0.0164	0.2653
rs3828962	vr133	G/A	5/103/583	5/92/363	0.0818	0.1109	1.0000	0.0251	0.3670
rs55950988	vr132	A/G	5/104/582	5/95/360	0.0825	0.1141	0.8187	0.0147	0.2439
rs2270314	vr131	C/G	5/107/579	6/97/357	0.0847	0.1185	1.0000	0.0090	0.1708
rs73169251	vr130	C/G	5/107/579	6/96/358	0.0847	0.1174	1.0000	0.0110	0.1977
rs73169250	vr129	T/C	5/107/579	6/96/358	0.0847	0.1174	1.0000	0.0110	0.1977
rs73169249	vr128	T/G	5/107/579	6/96/358	0.0847	0.1174	1.0000	0.0110	0.1977
rs72621132	vr127	G/A	18/172/501	17/142/301	0.1505	0.1913	1.0000	0.0081	0.1558
rs73169248	vr126	C/A	5/108/578	6/96/358	0.0854	0.1174	1.0000	0.0128	0.2177
rs73169245	vr125	C/G	6/110/575	6/103/351	0.0883	0.1250	0.8306	0.0058	0.1210
rs3793227	vr124	C/T	6/110/575	6/101/353	0.0883	0.1228	0.8301	0.0091	0.1711
rs73169239	vr123	T/G	5/111/575	5/97/358	0.0876	0.1163	0.8196	0.0255	0.3718
rs73169238	vr122	T/C	5/108/578	6/97/357	0.0854	0.1185	1.0000	0.0106	0.1916
rs73169234	vr121	A/G	5/108/578	6/96/358	0.0854	0.1174	1.0000	0.0128	0.2177
rs73169233	vr120	A/G	5/108/578	6/96/358	0.0854	0.1174	1.0000	0.0128	0.2177
rs55683317	vr119	A/G	5/108/578	6/96/358	0.0854	0.1174	1.0000	0.0128	0.2177
rs3793224	vr118	T/C	5/109/577	6/97/357	0.0861	0.1185	1.0000	0.0099	0.1825
rs56260213	vr117	A/G	5/110/576	6/96/358	0.0868	0.1174	1.0000	0.0146	0.2431
rs56023468	vr116	G/A	5/110/576	6/98/356	0.0868	0.1196	1.0000	0.0095	0.1766
rs55662704	vr115	G/A	5/110/576	6/98/356	0.0868	0.1196	1.0000	0.0095	0.1766
rs3793223	vr114	T/G	5/107/579	5/97/358	0.0847	0.1163	0.8196	0.0127	0.2168
rs3793222	vr113	A/C	5/107/579	5/97/358	0.0847	0.1163	0.8196	0.0127	0.2168
rs3815516	vr112	T/A	18/171/502	16/144/300	0.1498	0.1913	0.8810	0.0074	0.1476
rs75463077	vr111	A/G	3/74/614	3/67/390	0.0579	0.0794	1.0000	0.0491	0.5616

	Sequential	Alleles	Genotype counts (11/12/22) (N=1,151)		Minor allele freq.		HWE (Controls)	Association (additive, adjusted for sex & age)	
SNP*	No.*	(1/2)[@]	Cases (n=691)	Controls (n=460)	Cases	Controls	*P* value	P_a	P_{aemp}
rs1476998	vr110	G/A	18/172/501	16/144/300	0.1505	0.1913	0.8810	0.0074	0.1476
rs55864635	vr109	A/G	5/107/579	6/96/358	0.0847	0.1174	1.0000	0.0110	0.1975
rs3793220	vr108	T/C	5/125/561	4/105/351	0.0977	0.1228	0.2785	0.0586	0.6184
rs73523914	vr107	C/G	28/222/441	20/162/278	0.2012	0.2196	0.6828	0.2308	0.9745
rs114961653	vr106	C/T	4/111/576	2/105/353	0.0861	0.1185	0.0455	0.0122	0.2112
rs7787641	vr105	C/T	4/104/583	6/95/359	0.0810	0.1163	1.0000	0.0053	0.1139
rs2540345	vr104	G/A	5/103/583	7/95/358	0.0818	0.1185	0.8224	0.0039	0.0885
rs56375711	vr103	T/C	4/105/582	5/89/366	0.0818	0.1076	1.0000	0.0408	0.5045
rs3812302	vr102	C/G	16/164/511	15/140/305	0.1418	0.1848	1.0000	0.0051	0.1105
rs3793217	vr101	C/T	4/109/578	4/92/364	0.0847	0.1087	0.6335	0.0664	0.6615
rs2730260	vr100	C/A	17/207/467	13/134/313	0.1744	0.1739	0.8717	0.8910	1.0000
rs73169220	vr099	G/T	6/128/557	8/107/345	0.1013	0.1337	1.0000	0.0134	0.2267
rs2730265	vr098	G/A	35/255/401	27/168/265	0.2352	0.2413	1.0000	0.5386	1.0000
rs2730264	vr097	T/C	17/211/463	13/137/310	0.1773	0.1772	0.7499	0.8861	1.0000
rs56236179	vr096	T/C	6/128/557	8/108/344	0.1013	0.1348	1.0000	0.0105	0.1911
rs2540357	vr095	C/G	19/186/486	19/152/289	0.1621	0.2065	1.0000	0.0033	0.0764
rs73169218	vr094	T/A	6/126/559	8/105/347	0.0999	0.1315	1.0000	0.0116	0.2053
rs7784586	vr093	G/A	7/124/560	8/109/343	0.0999	0.1359	1.0000	0.0065	0.1335
rs2540359	vr092	C/T	7/126/558	8/109/343	0.1013	0.1359	1.0000	0.0087	0.1663
rs2730254	vr091	G/C	7/126/558	8/109/343	0.1013	0.1359	1.0000	0.0087	0.1663
rs73169216	vr090	T/C	6/126/559	8/104/348	0.0999	0.1304	0.8396	0.0173	0.2771
rs73169215	vr089	G/A	6/126/559	8/105/347	0.0999	0.1315	1.0000	0.0145	0.2413
rs886656	vr088	T/C	6/126/559	8/104/348	0.0999	0.1304	0.8396	0.0173	0.2771
rs2730224	vr087	T/C	7/129/555	7/110/343	0.1035	0.1348	0.6926	0.0176	0.2809
rs7797868	vr086	C/T	119/333/239	93/228/139	0.4132	0.4500	1.0000	0.0612	0.6343
rs1017032	vr085	C/A	19/199/473	13/136/311	0.1715	0.1761	0.8720	0.5266	1.0000
rs2071623	vr084	A/G	72/317/302	65/204/191	0.3336	0.3630	0.3662	0.0733	0.6928
rs2071624	vr083	C/T	69/318/304	65/206/189	0.3300	0.3652	0.4816	0.0326	0.4351
rs2071625	vr082	G/A	61/307/323	59/201/200	0.3104	0.3467	0.4712	0.0212	0.3218
rs10268921	vr081	G/A	61/307/323	60/199/201	0.3104	0.3467	0.3542	0.0208	0.3172
rs34621730	vr080	A/C	92/316/283	78/219/163	0.3618	0.4076	0.7723	0.0131	0.2207
rs115794009	vr079	T/C	3/103/585	6/80/374	0.0789	0.1000	0.4361	0.0531	0.5868
rs2730220	vr078	A/G	2/89/600	3/80/377	0.0673	0.0935	0.7846	0.0134	0.2269
rs1465227	vr077	T/C	3/103/585	6/80/374	0.0789	0.1000	0.4361	0.0531	0.5868
rs885861	vr076	T/C	14/194/483	14/125/321	0.1606	0.1663	0.6168	0.5272	1.0000
rs1062610	vr075	A/G	14/193/484	14/125/321	0.1599	0.1663	0.6168	0.4965	0.9999
rs885863	vr074	A/G	21/201/469	14/140/306	0.1758	0.1826	0.7564	0.6018	1.0000
rs78625053	vr073	A/G	3/103/585	6/82/372	0.0789	0.1022	0.4545	0.0356	0.4628
rs2301897	vr072	T/C	3/103/585	6/81/373	0.0789	0.1011	0.4448	0.0424	0.5153
rs2301898	vr071	G/C	92/316/283	82/214/164	0.3618	0.4109	0.4408	0.0116	0.2046
rs2730242	vr070	C/T	62/303/326	59/198/203	0.3090	0.3435	0.3519	0.0289	0.4045
rs6951304	vr069	C/T	3/103/585	6/81/373	0.0789	0.1011	0.4448	0.0424	0.5153
rs11972299	vr068	G/T	3/103/585	5/82/373	0.0789	0.1000	0.7949	0.0489	0.5601

SNP*	Sequential No.*	Alleles (1/2)[@]	Genotype counts (11/12/22) (N=1,151) Cases (n=691)	Controls (n=460)	Minor allele freq. Cases	Controls	HWE (Controls) *P* value	Association (additive, adjusted for sex & age) P_a	P_{aemp}
rs115079694	vr067	A/G	3/104/584	6/85/369	0.0796	0.1054	0.6206	0.0235	0.3492
rs12113517	vr066	G/A	34/247/410	30/165/265	0.2279	0.2446	0.5288	0.2036	0.9592
rs11769917	vr065	A/G	14/195/482	14/125/321	0.1614	0.1663	0.6168	0.6015	1.0000
rs2730206	vr064	G/C	13/194/484	13/126/321	0.1592	0.1652	0.8659	0.5411	1.0000
rs13221124	vr063	G/A	93/316/282	82/214/164	0.3632	0.4109	0.4408	0.0122	0.2113
rs79123584	vr062	A/G	3/103/585	6/80/374	0.0789	0.1000	0.4361	0.0531	0.5868
rs2428780	vr061	T/A	13/194/484	14/125/321	0.1592	0.1663	0.6168	0.4965	0.9999
rs78494323	vr060	T/C	10/179/502	12/103/345	0.1440	0.1380	0.2361	0.9015	1.0000
rs28485090	vr059	C/A	89/336/266	70/212/178	0.3719	0.3826	0.6218	0.3037	0.9951
rs1966360	vr058	T/C	14/193/484	14/125/321	0.1599	0.1663	0.6168	0.4965	0.9999
rs6964992	vr057	G/A	89/336/266	70/211/179	0.3719	0.3815	0.5539	0.3254	0.9967
rs6961005	vr056	G/A	90/335/266	71/210/179	0.3726	0.3826	0.4898	0.2967	0.9937
rs75760684	vr055	T/G	10/179/502	12/103/345	0.1440	0.1380	0.2361	0.9015	1.0000
rs56843161	vr054	A/G	10/179/502	12/103/345	0.1440	0.1380	0.2361	0.9015	1.0000
rs2040897	vr053	T/C	49/321/321	44/193/223	0.3032	0.3054	0.8262	0.6110	1.0000
rs61612978	vr052	G/C	11/181/499	13/104/343	0.1469	0.1413	0.1749	0.8977	1.0000
rs79393253	vr051	C/T	11/182/498	13/105/342	0.1476	0.1424	0.1786	0.9105	1.0000
rs2730250	vr050	T/C	49/321/321	44/193/223	0.3032	0.3054	0.8262	0.6110	1.0000
rs58684874	vr049	T/C	10/179/502	12/104/344	0.1440	0.1391	0.2406	0.9507	1.0000
rs77288867	vr048	A/G	10/179/502	12/104/344	0.1440	0.1391	0.2406	0.9507	1.0000
rs2527237	vr047	A/G	13/194/484	14/127/319	0.1592	0.1685	0.7396	0.4024	0.9994
rs2657340	vr046	C/T	49/319/323	44/193/223	0.3017	0.3054	0.8262	0.5556	1.0000
rs2527236	vr045	A/G	13/193/485	14/127/319	0.1585	0.1685	0.7396	0.3784	0.9989
rs2730274	vr044	T/G	49/319/323	44/192/224	0.3017	0.3043	0.7421	0.5883	1.0000
rs76425232	vr043	A/G	10/178/503	12/103/345	0.1433	0.1380	0.2361	0.9483	1.0000
rs73169203	vr042	A/G	173/337/181	101/224/135	0.4942	0.4630	0.7075	0.1093	0.8212
rs75734602	vr041	A/G	10/179/502	12/103/345	0.1440	0.1380	0.2361	0.9015	1.0000
rs2467810	vr040	C/T	31/219/441	25/153/282	0.2033	0.2207	0.4976	0.3714	0.9986
rs10237003	vr039	G/A	137/345/209	93/223/144	0.4479	0.4446	0.7060	0.8960	1.0000
rs2730228	vr038	G/A	20/206/465	23/139/298	0.1780	0.2011	0.1936	0.0737	0.6955
rs76749764	vr037	A/G	9/157/525	7/88/365	0.1266	0.1109	0.4795	0.3107	0.9954
rs2730230	vr036	G/A	22/227/442	20/139/301	0.1961	0.1946	0.4565	0.9885	1.0000
rs2657397	vr035	A/G	34/221/436	30/154/276	0.2091	0.2326	0.1913	0.2208	0.9707
rs2730231	vr034	G/A	33/227/431	24/144/292	0.2120	0.2087	0.2598	0.8415	1.0000
rs2657398	vr033	G/A	33/223/435	30/154/276	0.2091	0.2326	0.1913	0.2177	0.9691
rs2657399	vr032	T/G	25/252/414	23/154/283	0.2185	0.2174	0.7838	0.9451	1.0000
rs2730232	vr031	G/A	62/253/376	46/182/232	0.2728	0.2978	0.2646	0.1735	0.9340
rs2657404	vr030	T/C	27/199/465	22/149/289	0.1831	0.2098	0.6726	0.1628	0.9212
rs73167296	vr029	G/A	127/335/229	97/218/145	0.4262	0.4478	0.3961	0.1957	0.9537
rs2657405	vr028	A/C	44/246/401	32/155/273	0.2417	0.2380	0.1243	0.8011	1.0000
rs73167295	vr027	A/T	125/337/229	97/217/146	0.4247	0.4467	0.3456	0.1988	0.9551
rs2657408	vr026	C/T	5/143/543	7/106/347	0.1107	0.1304	1.0000	0.1006	0.7970
rs78367179	vr025	G/C	12/187/492	12/106/342	0.1527	0.1413	0.2535	0.6171	1.0000

SNP*	Sequential No.*	Alleles (1/2)@	Genotype counts (11/12/22) (N=1,151) Cases (n=691)	Controls (n=460)	Minor allele freq. Cases	Controls	HWE (Controls) *P* value	Association (additive, adjusted for sex & age) P_a	P_{aemp}
rs7807501	vr024	T/C	12/187/492	12/106/342	0.1527	0.1413	0.2535	0.6171	1.0000
rs262149	vr023	A/G	30/221/440	27/152/281	0.2033	0.2239	0.2846	0.3242	0.9966
rs58655106	vr022	G/T	12/187/492	12/106/342	0.1527	0.1413	0.2535	0.6171	1.0000
rs56997940	vr021	C/T	12/187/492	12/106/342	0.1527	0.1413	0.2535	0.6171	1.0000
rs262150	vr020	T/C	31/220/440	27/152/281	0.2041	0.2239	0.2846	0.3242	0.9966
rs79160555	vr019	C/T	12/187/492	12/107/341	0.1527	0.1424	0.3370	0.6608	1.0000
rs262154	vr018	T/C	31/220/440	27/152/281	0.2041	0.2239	0.2846	0.3242	0.9966
rs6459916	vr017	C/T	48/271/372	43/195/222	0.2656	0.3054	1.0000	0.0408	0.5047
rs6956589	vr016	A/G	28/215/448	26/144/290	0.1961	0.2130	0.1638	0.4086	0.9997
rs55882200	vr015	G/C	154/327/210	93/217/150	0.4595	0.4380	0.3939	0.2581	0.9829
rs55974535	vr014	C/G	157/326/208	95/216/149	0.4631	0.4413	0.2998	0.2807	0.9902
rs35113968	vr013	C/T	34/229/428	27/158/275	0.2149	0.2304	0.5110	0.4428	0.9999
rs2730249	vr012	A/G	34/229/428	28/158/274	0.2149	0.2326	0.4331	0.3714	0.9986
rs75341666	vr011	A/G	2/92/597	5/68/387	0.0695	0.0848	0.3571	0.1052	0.8098
rs2527220	vr010	G/A	2/91/598	5/75/380	0.0687	0.0924	0.5730	0.0190	0.2968
rs75774480	vr009	T/C	2/86/603	5/65/390	0.0651	0.0815	0.2103	0.0839	0.7370
rs114886265	vr008	A/G	1/85/605	5/64/391	0.0630	0.0804	0.2000	0.0618	0.6381
rs2657326	vr007	A/G	50/284/357	45/196/219	0.2779	0.3109	0.9135	0.0783	0.7153
rs114906121	vr006	G/A	2/87/602	5/66/389	0.0659	0.0826	0.2215	0.0830	0.7342
rs78043053	vr005	T/C	2/87/602	5/66/389	0.0659	0.0826	0.2215	0.0804	0.7237
rs262136	vr004	A/G	2/92/597	5/75/380	0.0695	0.0924	0.5730	0.0234	0.3474
rs6948415	vr003	C/T	2/89/600	5/68/387	0.0673	0.0848	0.3571	0.0759	0.7055
rs2657389	vr002	T/C	35/227/429	27/160/273	0.2149	0.2326	0.6013	0.3421	0.9976
rs262134	vr001	G/A	63/290/338	52/218/190	0.3010	0.3500	0.4129	0.0104	0.1890

* The single-nucleotide polymorphisms (SNPs) are tabulated from top to bottom in the order from the 5' end to the 3' end of the sense strand (i.e. the minus strand of chromosome 7) of the *VIPR2* gene while the sequential number is based on the chromosomal position on the plus strand.

@ The minor allele is coded as allele 1, and the major allele as allele 2. The bases of the alleles are coded based on the sense strand of VIPR2 (i.e. the minus strand of chromosome 7).

Supplementary Table 2. Stage-1 study: Summary of significant omnibus tests for sliding-window haplotype analysis of 152 *VIPR2* single-nucleotide polymorphisms (SNPs)*

**vr107** (rs73523914)	**vr106** rs114961653)	**vr105** (rs7787641)	**vr104** (rs2540345)	**vr103** (rs56375711)	**vr102** (rs3812302)	**vr101** (rs3793217)	**vr100** (rs2730260)	**vr099** (rs73169220)	**vr098** (rs2730265)	**vr097** (rs2730264)	**vr096** (rs56236179)	**vr095** (rs2540357)	**vr094** (rs73169218)	**vr093** (rs7784586)	**vr092** (rs2540359)	**vr091** (rs2730254)	**vr090** (rs73169216)	**vr089** (rs73169215)	**vr088** (rs886656)	**vr087** (rs2730224)	Size of sliding window (no. of SNPs)	Haplotype analysis (Omnibus test)	
																						P_a value	P_{aemp} value
										■	■										2	9.02×10^{-4}	0.0435
						■	■	■													3	1.03×10^{-3}	0.5000
										■	■	■									3	7.23×10^{-4}	0.0358
									■	■	■	■									4	4.42×10^{-4}	0.0231
										■	■	■	■								4	7.59×10^{-4}	0.0374
								■	■	■	■	■									5	4.42×10^{-4}	0.0231
									■	■	■	■	■								5	4.88×10^{-4}	0.0250
										■	■	■	■	■							5	4.25×10^{-4}	0.0222
			■	■	■	■	■	■													6	7.81×10^{-4}	0.0384
							■	■	■	■	■	■									6	5.35×10^{-4}	0.0280
								■	■	■	■	■	■								6	4.88×10^{-4}	0.0250
									■	■	■	■	■	■							6	4.82×10^{-4}	0.0243
										■	■	■	■	■	■						6	4.25×10^{-4}	0.0222
		■	■	■	■	■	■	■													7	8.29×10^{-4}	0.0410
						■	■	■	■	■	■	■									7	7.65×10^{-4}	0.0376
							■	■	■	■	■	■	■								7	6.37×10^{-4}	0.0323
								■	■	■	■	■	■	■							7	4.82×10^{-4}	0.0243
									■	■	■	■	■	■	■						7	4.82×10^{-4}	0.0243
										■	■	■	■	■	■	■					7	4.25×10^{-4}	0.0222
	■	■	■	■	■	■	■	■													8	8.58×10^{-4}	0.0422
						■	■	■	■	■	■	■	■								8	8.24×10^{-4}	0.0410
							■	■	■	■	■	■	■	■							8	6.37×10^{-4}	0.0323
								■	■	■	■	■	■	■	■						8	4.82×10^{-4}	0.0243
									■	■	■	■	■	■	■	■					8	4.82×10^{-4}	0.0243
										■	■	■	■	■	■	■	■				8	5.27×10^{-4}	0.0269
■	■	■	■	■	■	■	■	■													9	1.06×10^{-3}	0.0500
						■	■	■	■	■	■	■	■	■							9	8.24×10^{-4}	0.0410
							■	■	■	■	■	■	■	■	■						9	6.37×10^{-4}	0.0323
								■	■	■	■	■	■	■	■	■					9	4.82×10^{-4}	0.0243
									■	■	■	■	■	■	■	■	■				9	5.94×10^{-4}	0.0299
										■	■	■	■	■	■	■	■	■			9	5.27×10^{-4}	0.0269
						■	■	■	■	■	■	■	■	■	■						10	8.24×10^{-4}	0.0410
							■	■	■	■	■	■	■	■	■	■					10	6.37×10^{-4}	0.0323
								■	■	■	■	■	■	■	■	■	■				10	5.94×10^{-4}	0.0299
									■	■	■	■	■	■	■	■	■	■			10	5.94×10^{-4}	0.0299
										■	■	■	■	■	■	■	■	■	■		10	5.27×10^{-4}	0.0269
						■	■	■	■	■	■	■	■	■	■	■					11	8.24×10^{-4}	0.0410
							■	■	■	■	■	■	■	■	■	■	■				11	7.81×10^{-4}	0.0384
								■	■	■	■	■	■	■	■	■	■	■			11	5.94×10^{-4}	0.0299
									■	■	■	■	■	■	■	■	■	■	■		11	5.94×10^{-4}	0.0299
										■	■	■	■	■	■	■	■	■	■	■	11	6.83×10^{-4}	0.0340
						■	■	■	■	■	■	■	■	■	■	■	■				12	9.33×10^{-4}	0.0451
							■	■	■	■	■	■	■	■	■	■	■	■			12	7.81×10^{-4}	0.0384
								■	■	■	■	■	■	■	■	■	■	■	■		12	5.94×10^{-4}	0.0299
									■	■	■	■	■	■	■	■	■	■	■	■	12	7.68×10^{-4}	0.0377
						■	■	■	■	■	■	■	■	■	■	■	■	■			13	9.33×10^{-4}	0.0451
							■	■	■	■	■	■	■	■	■	■	■	■	■		13	7.81×10^{-4}	0.0384
								■	■	■	■	■	■	■	■	■	■	■	■	■	13	7.68×10^{-4}	0.0377
						■	■	■	■	■	■	■	■	■	■	■	■	■	■		14	9.33×10^{-4}	0.0451
							■	■	■	■	■	■	■	■	■	■	■	■	■	■	14	1.00×10^{-3}	0.0481

* In Stage-1 study, 152 SNPs were genotyped. Sliding-window haplotype analysis was performed with window size ranging from 1 to 15 SNPs per window. There were a total of 2,175 sliding windows. Of these, only 50 sliding windows remained statistically significant even after multiple-testing correction by permutation tests (10,000 permutations) as shown above. Shaded boxes below header row indicate the SNPs constituting the significant sliding windows.

Supplementary Table 3. Stage-2 study: Summary of *VIPR2* genotype data and single-marker association testing

SNP*	Sequential No.*	Alleles (1/2)@	Genotype counts (11/12/22) (N=636) Cases (n=250)	Controls (n=386)	Minor allele freq. Cases	Controls	HWE (Controls) *P* value	Association (additive, adjusted for sex & age) P_a	P_{aemp}
rs73523914	vr107	C/G	17/80/153	24/133/229	0.2280	0.2345	0.4776	0.3943	0.9016
rs114961653	vr106	C/T	3/46/201	9/86/291	0.1040	0.1347	0.3813	0.0550	0.2341
rs7787641	vr105	C/T	2/45/203	9/82/295	0.0980	0.1295	0.2572	0.0500	0.2139
rs2540345	vr104	G/A	2/51/197	9/82/295	0.1100	0.1295	0.2572	0.1549	0.5133
rs56375711	vr103	T/C	2/48/200	9/79/298	0.1040	0.1256	0.1674	0.1205	0.4250
s3812302	vr102	C/G	8/67/175	16/114/256	0.1660	0.1891	0.5056	0.1629	0.5312
rs3793217	vr101	C/T	1/46/203	9/85/292	0.0960	0.1334	0.3751	0.0290	0.1333
rs2730260	vr100	C/A	7/83/160	15/121/250	0.1940	0.1956	1.0000	0.8164	1.0000
rs73169220	vr099	G/T	1/54/195	13/100/273	0.1120	0.1632	0.3490	0.0093	0.0492
rs2730265	vr098	G/A	15/101/134	30/135/221	0.2620	0.2526	0.1406	0.7326	0.9998
rs2730264	vr097	T/C	5/91/154	16/126/244	0.2020	0.2047	1.0000	0.8915	1.0000
rs56236179	vr096	T/C	1/62/187	13/101/272	0.1280	0.1645	0.3536	0.0574	0.2416
rs2540357	vr095	C/G	9/77/164	21/126/239	0.1900	0.2176	0.4539	0.1281	0.4438
rs73169218	vr094	T/A	1/55/194	12/96/278	0.1140	0.1554	0.3297	0.0266	0.1235
rs7784586	vr093	G/A	3/54/193	12/98/276	0.1200	0.1580	0.3420	0.0436	0.1913
rs2540359	vr092	C/T	3/54/193	12/98/276	0.1200	0.1580	0.3420	0.0522	0.2228
rs2730254	vr091	G/C	3/56/191	12/98/276	0.1240	0.1580	0.3420	0.0915	0.3430
rs73169216	vr090	T/C	1/55/194	12/97/277	0.1140	0.1567	0.3350	0.0242	0.1130
rs73169215	vr089	G/A	1/55/194	12/97/277	0.1140	0.1567	0.3350	0.0242	0.1130
rs886656	vr088	T/C	2/57/191	12/97/277	0.1220	0.1567	0.3350	0.0651	0.2666
rs2730224	vr087	T/C	3/56/191	12/100/274	0.1240	0.1606	0.4493	0.0564	0.2378

* The single-nucleotide polymorphisms (SNPs) are tabulated from top to bottom in the order from the 5’ end to the 3’ end of the sense strand (i.e. the minus strand of chromosome 7) of the *VIPR2* gene while the sequential number is based on the chromosomal position on the plus strand.

@ The minor allele is coded as allele 1, and the major allele as allele 2. The bases of the alleles are coded based on the sense strand of *VIPR2* (i.e. the minus strand of chromosome 7).

Supplementary Table 4. Stage-2 study: Summary of significant omnibus tests for sliding-window haplotype analysis of 21 *VIPR2* single-nucleotide polymorphisms (SNPs)*

**vr107** (rs73523914)	**vr106** rs114961653)	**vr105** (rs7787641)	**vr104** (rs2540345)	**vr103** (rs56375711)	**vr102** (rs3812302)	**vr101** (rs3793217)	**vr100** (rs2730260)	**vr099** (rs73169220)	**vr098** (rs2730265)	**vr097** (rs2730264)	**vr096** (rs56236179)	**vr095** (rs2540357)	**vr094** (rs73169218)	**vr093** (rs7784586)	**vr092** (rs2540359)	**vr091** (rs2730254)	**vr090** (rs73169216)	**vr089** (rs73169215)	**vr088** (rs886656)	**vr087** (rs2730224)	Size of sliding window (no. of SNPs)	Haplotype analysis (Omnibus test)	
																						P_a value	P_{aemp} value
							■	■													2	0.0002	0.0034
								■	■												2	0.0024	0.0246
										■	■										2	0.0031	0.0307
						■	■	■													3	0.0041	0.0387
							■	■	■												3	0.0033	0.0325
								■	■	■											3	0.0022	0.0216
					■	■	■	■													4	0.0053	0.0499
										■	■	■	■								4	0.0045	0.0426
										■	■	■	■	■							5	0.0045	0.0422
							■	■	■	■	■	■	■								7	0.0051	0.0490
									■	■	■	■	■	■	■						7	0.0041	0.0385
										■	■	■	■	■	■	■					7	0.0036	0.0349
	■	■	■	■	■	■	■	■													8	0.0035	0.0337
							■	■	■	■	■	■	■	■							8	0.0050	0.0481
										■	■	■	■	■	■	■	■				8	0.0036	0.0349
							■	■	■	■	■	■	■	■	■						9	0.0050	0.0485
										■	■	■	■	■	■	■	■	■			9	0.0036	0.0349
										■	■	■	■	■	■	■	■	■	■		10	0.0037	0.0349
										■	■	■	■	■	■	■	■	■	■	■	11	0.0037	0.0349

* In Stage-2 study, 20 SNPs were genotyped and the genotypes of one SNP (vr106) imputed. Sliding-window haplotype analysis was performed with window size ranging from 1 to 15 SNPs per window. There were a total of 210 sliding windows. Of these, only 19 sliding windows remained statistically significant even after multiple-testing correction by permutation tests (10,000 permutations) as shown above. Shaded boxes below header row indicate the SNPs constituting the significant sliding windows.

Supplementary Table 5. Stage-3 study: summary of *VIPR2* genotype data and single-marker association testing*

SNP*	Sequential No.	Alleles (1/2)	Genotype counts (11/12/22) (N=1,787)		Minor allele freq.		HWE (Controls)	Association (additive, adjusted for sex & age)		
			Cases (n=941)	Controls (n=846)	Cases	Controls	*P* value	OR	P_a	P_{aemp}
rs73523914	vr107	C/G	45/302/594	44/295/507	0.2083	0.2264	0.9218	0.8747	0.1038	0.3722
rs114961653	vr106	C/T	7/157/777	11/191/644	0.0909	0.1259	0.5336	0.6755	0.0004	0.0027
rs7787641	vr105	C/T	6/149/786	15/177/654	0.0856	0.1223	0.4248	0.6581	0.0002	0.0016
rs2540345	vr104	G/A	7/154/780	16/177/653	0.0893	0.1235	0.3390	0.6786	0.0005	0.0028
rs56375711	vr103	T/C	6/153/782	14/168/664	0.0877	0.1158	0.3990	0.7163	0.0031	0.0187
rs3812302	vr102	C/G	24/231/686	31/254/561	0.1482	0.1868	0.7343	0.7438	0.0011	0.0078
rs3793217	vr101	C/T	5/155/781	13/177/656	0.0877	0.1200	0.7453	0.6959	0.0013	0.0089
rs2730260	vr100	C/A	24/290/627	28/255/563	0.1796	0.1838	1.0000	0.9661	0.7001	0.9994
rs73169220	vr099	G/T	7/182/752	21/207/618	0.1041	0.1472	0.4923	0.6551	0.0001	0.0003
rs2730265	vr098	G/A	50/356/535	57/303/486	0.2423	0.2465	0.3085	0.9615	0.6203	0.9946
rs2730264	vr097	T/C	22/302/617	29/263/554	0.1838	0.1897	0.8233	0.9539	0.5973	0.9917
rs56236179	vr096	T/C	7/190/744	21/209/616	0.1084	0.1483	0.4971	0.6752	0.0002	0.0010
rs2540357	vr095	C/G	28/263/650	40/278/528	0.1695	0.2116	0.6800	0.7348	0.0004	0.0027
rs73169218	vr094	T/A	7/181/753	20/201/625	0.1036	0.1424	0.4000	0.6711	0.0002	0.0011
rs7784586	vr093	G/A	10/178/753	20/207/619	0.1052	0.1460	0.5811	0.6720	0.0001	0.0009
rs2540359	vr092	C/T	10/180/751	20/207/619	0.1063	0.1460	0.5811	0.6786	0.0002	0.0015
rs2730254	vr091	G/C	10/182/749	20/207/619	0.1073	0.1460	0.5811	0.6877	0.0003	0.0022
rs73169216	vr090	T/C	7/181/753	20/201/625	0.1036	0.1424	0.4000	0.6753	0.0002	0.0016
rs73169215	vr089	G/A	7/181/753	20/202/624	0.1036	0.1430	0.4819	0.6721	0.0002	0.0012
rs886656	vr088	T/C	8/183/750	20/201/625	0.1057	0.1424	0.4000	0.6905	0.0004	0.0026
rs2730224	vr087	T/C	10/185/746	19/210/617	0.1089	0.1466	0.7840	0.6918	0.0004	0.0026

* Stage-3 study combined the genotype data for 21 SNPs from the first two stages. Therefore, the total sample size is 1,787 subjects. The single-nucleotide polymorphisms (SNPs) are tabulated from top to bottom in the order from the 5’ end to the 3’ end of the sense strand (i.e. the minus strand of chromosome 7) of the *VIPR2* gene while the sequential number is based on the chromosomal position on the plus strand. The minor allele is coded as allele 1, and the major allele as allele 2. The bases of the alleles are coded based on the sense strand of *VIPR2* (i.e. the minus strand of chromosome 7).

Supplementary Table 6. Summary of sliding-window haplotype analysis of the entire sample (Stage 3): Omnibus tests and significant haplotypes*

Omnibus test (degree of freedom = H - 1)						Significant haplotype in the sliding window shown on the left						
Sliding window						Significant haplotype (*VIPR2* sense strand, 5'>3'; major allele in blue and minor allele in red)	Haplotype frequency in					
No. of SNPs per window	No. of common haplotypes (H; MHF>0.01)	First SNP	Last SNP	Asymptotic *P* value (P_a)	Empirical *P* value (P_{aemp})		Entire sample	Cases	Controls	Odds ratio	Asymptotic *P* value (P_a)	Empirical *P* value (P_{aemp})
						**High-risk haplotypes (odds ratio >1.00)**						
1	2	vr107	vr107	0.1040	0.5188	G	0.7830	0.7925	0.7741	1.14	0.1040	0.7975
2	4	vr107	vr106	0.0033	0.0329	GT	0.7680	0.7817	0.7535	1.20	0.0234	0.3407
3	4	vr107	vr105	0.0020	0.0215	GTT	0.7660	0.7883	0.7604	1.20	0.0226	0.3325
4	4	vr107	vr104	0.0032	0.0324	GTTA	0.7640	0.7901	0.7612	1.20	0.0264	0.3713
5	4	vr107	vr103	0.0066	0.0614	GTTAC	0.7620	0.7897	0.7642	1.18	0.0372	0.4662
6	5	vr107	vr102	0.0032	0.0321	GTTACG	0.7550	0.7897	0.7623	1.23	0.0084	0.1548
7	4	vr107	vr101	0.0013	0.0143	GTTACGT	0.7550	0.8005	0.7839	1.24	0.0074	0.1398
8	5	vr107	vr100	0.0001	0.0015	GTTACGT C	0.0675	0.0855	0.0556	1.65	0.0004	0.0102
9	6	vr107	vr099	4.53E-06	7.00E-05	GTTACGT CT	0.0517	0.0710	0.0365	2.15	5.44E-06	0.0001
10	6	vr107	vr098	1.96E-05	0.0003	GTTACGT CT G	0.0531	0.0725	0.0386	2.06	1.05E-05	0.0002
11	6	vr107	vr097	3.31E-05	0.0005	GTTACGT CT GT	0.0528	0.0720	0.0385	2.04	1.31E-05	0.0003
12	6	vr107	vr096	6.37E-05	0.0010	GTTACGT CT GTC	0.0522	0.0710	0.0385	2.00	2.27E-05	0.0006
13	6	vr107	vr095	6.36E-05	0.0010	GTTACGT CT GTCG	0.0522	0.0711	0.0385	2.01	2.18E-05	0.0006
14	6	vr107	vr094	7.21E-05	0.0010	GTTACGT CT GTCGA	0.0522	0.0712	0.0385	2.01	2.18E-05	0.0006
15	6	vr107	vr093	7.88E-05	0.0011	GTTACGT CT GTCGAA	0.0522	0.0712	0.0385	2.01	2.18E-05	0.0006
1	2	vr106	vr106	0.0004	0.0052	T	0.8930	0.9091	0.8743	1.48	0.0004	0.0108
2	2	vr106	vr105	0.0003	0.0040	TT	0.8900	0.9158	0.8801	1.48	0.0003	0.0081
3	2	vr106	vr104	0.0007	0.0078	TTA	0.8860	0.9169	0.8809	1.44	0.0007	0.0164
4	2	vr106	vr103	0.0015	0.0163	TTAC	0.8850	0.9167	0.8846	1.40	0.0015	0.0332
5	3	vr106	vr102	0.0046	0.0449	TTACG	0.8250	0.8587	0.8240	1.31	0.0025	0.0548
6	3	vr106	vr101	0.0019	0.0202	TTACGT	0.8200	0.8616	0.8305	1.33	0.0011	0.0249
7	4	vr106	vr100	1.24E-05	0.0002	TTACGT C	0.0656	0.0849	0.0502	1.77	6.35E-05	0.0015
8	5	vr106	vr099	8.87E-07	2.00E-05	TTACGT CT	0.0503	0.0705	0.0326	2.30	**1.03E-06**	2.00E-05
9	5	vr106	vr098	5.38E-06	0.0001	TTACGT CT G	0.0547	0.0747	0.0383	2.11	3.84E-06	8.00E-05
10	5	vr106	vr097	9.35E-06	0.0002	TTACGT CT GT	0.0544	0.0742	0.0383	2.09	4.92E-06	0.0001
11	5	vr106	vr096	2.65E-05	0.0004	TTACGT CT GTC	0.0536	0.0727	0.0383	2.04	1.11E-05	0.0003
12	5	vr106	vr095	2.71E-05	0.0004	TTACGT CT GTCG	0.0536	0.0728	0.0383	2.04	1.11E-05	0.0003
13	5	vr106	vr094	3.10E-05	0.0004	TTACGT CT GTCGA	0.0536	0.0729	0.0383	2.04	1.11E-05	0.0003
14	5	vr106	vr093	3.42E-05	0.0005	TTACGT CT GTCGAA	0.0536	0.0730	0.0384	2.04	1.11E-05	0.0003
15	5	vr106	vr092	3.26E-05	0.0005	TTACGT CT GTCGAAT	0.0536	0.0730	0.0384	2.03	1.14E-05	0.0003
1	2	vr105	vr105	0.0002	0.0026	T	0.8970	0.9144	0.8778	1.52	0.0002	0.0049
2	2	vr105	vr104	0.0006	0.0073	TA	0.8930	0.9155	0.8786	1.46	0.0006	0.0154
3	2	vr105	vr103	0.0014	0.0158	TAC	0.8910	0.9157	0.8833	1.42	0.0014	0.0317
4	3	vr105	vr102	0.0033	0.0330	TACG	0.8270	0.8550	0.8174	1.33	0.0014	0.0325
5	3	vr105	vr101	0.0015	0.0169	TACGT	0.8200	0.8575	0.8236	1.35	0.0007	0.0167
6	4	vr105	vr100	3.97E-05	0.0006	TACGT C	0.0694	0.0870	0.0553	1.68	0.0002	0.0054
7	5	vr105	vr099	2.19E-06	5.00E-05	TACGT CT	0.0531	0.0720	0.0363	2.17	3.28E-06	7.00E-05
8	5	vr105	vr098	9.52E-06	0.0002	TACGT CT G	0.0543	0.0734	0.0383	2.07	6.63E-06	0.0001
9	5	vr105	vr097	1.63E-05	0.0003	TACGT CT GT	0.0539	0.0730	0.0382	2.05	8.38E-06	0.0002
10	5	vr105	vr096	1.66E-05	0.0003	TACGT CT GTC	0.0539	0.0730	0.0382	2.05	9.14E-06	0.0002
11	5	vr105	vr095	9.24E-06	0.0002	TACGT CT GTCG	0.0488	0.0686	0.0328	2.22	3.48E-06	7.00E-05
12	5	vr105	vr094	9.98E-06	0.0002	TACGT CT GTCGA	0.0488	0.0688	0.0329	2.22	3.47E-06	7.00E-05
13	5	vr105	vr093	1.93E-05	0.0003	TACGT CT GTCGAA	0.0491	0.0689	0.0335	2.17	5.54E-06	0.0001
14	5	vr105	vr092	1.86E-05	0.0003	TACGT CT GTCGAAT	0.0491	0.0688	0.0335	2.16	5.69E-06	0.0001
15	5	vr105	vr091	3.16E-05	0.0005	TACGT CT GTCGAATC	0.0488	0.0683	0.0335	2.14	8.12E-06	0.0002
1	2	vr104	vr104	0.0005	0.0055	A	0.8950	0.9107	0.8766	1.47	0.0005	0.0113
2	2	vr104	vr103	0.0016	0.0177	AC	0.8920	0.9153	0.8834	1.41	0.0016	0.0361
3	3	vr104	vr102	0.0033	0.0328	ACG	0.8280	0.8546	0.8165	1.33	0.0013	0.0290
4	3	vr104	vr101	0.0014	0.0156	ACGT	0.8200	0.8571	0.8231	1.35	0.0006	0.0144
5	4	vr104	vr100	4.27E-05	0.0007	ACGT C	0.0695	0.0868	0.0557	1.66	0.0003	0.0069
6	5	vr104	vr099	2.61E-06	5.00E-05	ACGT CT	0.0531	0.0718	0.0366	2.16	4.11E-06	0.0001
7	5	vr104	vr098	9.28E-06	0.0002	ACGT CT G	0.0543	0.0735	0.0383	2.07	6.48E-06	0.0001
8	5	vr104	vr097	1.58E-05	0.0003	ACGT CT GT	0.0540	0.0730	0.0382	2.06	8.17E-06	0.0002
9	5	vr104	vr096	2.16E-05	0.0003	ACGT CT GTC	0.0537	0.0725	0.0382	2.04	1.10E-05	0.0003
10	5	vr104	vr095	1.82E-05	0.0003	ACGT CT GTCG	0.0536	0.0726	0.0381	2.04	9.91E-06	0.0002
11	5	vr104	vr094	2.07E-05	0.0003	ACGT CT GTCGA	0.0536	0.0727	0.0381	2.04	1.00E-05	0.0002
12	5	vr104	vr093	2.29E-05	0.0003	ACGT CT GTCGAA	0.0536	0.0728	0.0381	2.04	1.01E-05	0.0002
13	5	vr104	vr092	2.18E-05	0.0003	ACGT CT GTCGAAT	0.0536	0.0727	0.0382	2.04	1.03E-05	0.0002
14	5	vr104	vr091	3.77E-05	0.0006	ACGT CT GTCGAATC	0.0533	0.0723	0.0382	2.02	1.46E-05	0.0003
15	5	vr104	vr090	4.92E-05	0.0007	ACGT CT GTCGAATCC	0.0533	0.0724	0.0382	2.02	1.46E-05	0.0003
1	2	vr103	vr103	0.0031	0.0317	C	0.8990	0.9123	0.8838	1.40	0.0031	0.0660
2	3	vr103	vr102	0.0037	0.0376	CG	0.8310	0.8526	0.8130	1.34	0.0011	0.0257
3	3	vr103	vr101	0.0008	0.0088	CGT	0.8230	0.8548	0.8228	1.37	0.0004	0.0099
4	4	vr103	vr100	2.96E-05	0.0004	CGT C	0.0700	0.0866	0.0562	1.65	0.0004	0.0088
5	5	vr103	vr099	7.08E-07	2.00E-05	CGT CT	0.0535	0.0722	0.0364	2.19	2.71E-06	5.00E-05
6	5	vr103	vr098	3.18E-06	6.00E-05	CGT CT G	0.0549	0.0742	0.0383	2.10	4.16E-06	0.0001
7	5	vr103	vr097	7.34E-06	0.0001	CGT CT GT	0.0545	0.0735	0.0381	2.08	5.71E-06	0.0001

Omnibus test (degree of freedom = H - 1)						Significant haplotype in the sliding window shown on the left						
Sliding window						Significant haplotype (*VIPR2* sense strand, 5'>3'; major allele in blue and minor allele in red)	Haplotype frequency in					
No. of SNPs per window	No. of common haplotypes (H; MHF>0.01)	First SNP	Last SNP	Asymptotic *P* value (P_a)	Empirical *P* value (P_{aemp})		Entire sample	Cases	Controls	Odds ratio	Asymptotic *P* value (P_a)	Empirical *P* value (P_{aemp})
8	5	vr103	vr096	1.44E-05	0.0002	CGT CT GTC	0.0539	0.0725	0.0381	2.04	9.94E-06	0.0002
9	5	vr103	vr095	1.38E-05	0.0002	CGT CT GTCG	0.0538	0.0725	0.0379	2.05	9.13E-06	0.0002
10	5	vr103	vr094	1.53E-05	0.0003	CGT CT GTCGA	0.0538	0.0726	0.0380	2.05	9.13E-06	0.0002
11	5	vr103	vr093	1.70E-05	0.0003	CGT CT GTCGAA	0.0538	0.0728	0.0380	2.05	9.13E-06	0.0002
12	5	vr103	vr092	1.61E-05	0.0003	CGT CT GTCGAAT	0.0537	0.0727	0.0381	2.05	9.39E-06	0.0002
13	5	vr103	vr091	2.81E-05	0.0004	CGT CT GTCGAATC	0.0535	0.0722	0.0381	2.02	1.32E-05	0.0003
14	5	vr103	vr090	3.72E-05	0.0006	CGT CT GTCGAATCC	0.0535	0.0724	0.0381	2.02	1.32E-05	0.0003
15	5	vr103	vr089	3.72E-05	0.0006	CGT CT GTCGAATCCA	0.0535	0.0724	0.0381	2.02	1.32E-05	0.0003
1	2	vr102	vr102	0.0011	0.0130	G	0.8340	0.8529	0.8135	1.34	0.0011	0.0264
2	3	vr102	vr101	0.0013	0.0147	GT	0.8240	0.8520	0.8114	1.38	0.0004	0.0096
3	5	vr102	vr100	3.66E-05	0.0006	GT C	0.0712	0.0863	0.0567	1.62	0.0006	0.0135
4	5	vr102	vr099	4.31E-07	2.00E-05	GT CT	0.0538	0.0725	0.0363	2.20	2.26E-06	4.00E-05
5	5	vr102	vr098	1.62E-06	4.00E-05	GT CT G	0.0552	0.0745	0.0383	2.11	3.52E-06	7.00E-05
6	5	vr102	vr097	3.37E-06	6.00E-05	GT CT GT	0.0547	0.0739	0.0380	2.10	4.35E-06	0.0001
7	5	vr102	vr096	8.37E-06	0.0002	GT CT GTC	0.0541	0.0728	0.0380	2.05	8.03E-06	0.0002
8	5	vr102	vr095	8.03E-06	0.0002	GT CT GTCG	0.0540	0.0729	0.0378	2.06	7.30E-06	0.0002
9	5	vr102	vr094	8.95E-06	0.0002	GT CT GTCGA	0.0540	0.0730	0.0379	2.06	7.30E-06	0.0002
10	5	vr102	vr093	1.01E-05	0.0002	GT CT GTCGAA	0.0540	0.0731	0.0379	2.06	7.40E-06	0.0002
11	5	vr102	vr092	1.04E-05	0.0002	GT CT GTCGAAT	0.0540	0.0731	0.0379	2.06	7.30E-06	0.0002
12	5	vr102	vr091	1.84E-05	0.0003	GT CT GTCGAATC	0.0537	0.0727	0.0379	2.04	1.03E-05	0.0002
13	5	vr102	vr090	2.46E-05	0.0004	GT CT GTCGAATCC	0.0537	0.0728	0.0379	2.04	1.03E-05	0.0002
14	5	vr102	vr089	2.46E-05	0.0004	GT CT GTCGAATCCA	0.0537	0.0728	0.0379	2.04	1.03E-05	0.0002
15	5	vr102	vr088	2.22E-05	0.0003	GT CT GTCGAATCCAC	0.0537	0.0728	0.0379	2.04	1.03E-05	0.0002
1	2	vr101	vr101	0.0013	0.0150	T	0.8970	0.9118	0.8802	1.44	0.0013	0.0302
2	3	vr101	vr100	0.0007	0.0080	T C	0.0832	0.0943	0.0714	1.36	0.0132	0.2229
3	4	vr101	vr099	4.47E-07	2.00E-05	T CT	0.0596	0.0768	0.0415	1.94	1.16E-05	0.0003
4	5	vr101	vr098	2.88E-06	5.00E-05	T CT G	0.0590	0.0759	0.0415	1.92	1.71E-05	0.0005
5	5	vr101	vr097	6.48E-06	0.00014	T CT GT	0.0585	0.0756	0.0415	1.91	2.08E-05	0.0006
6	5	vr101	vr096	8.28E-06	0.00015	T CT GTC	0.0582	0.0751	0.0415	1.90	2.74E-05	0.0007
7	5	vr101	vr095	2.22E-06	5.00E-05	T CT GTCG	0.0540	0.0719	0.0370	2.06	7.02E-06	0.0002
8	5	vr101	vr094	2.79E-06	5.00E-05	T CT GTCGA	0.0540	0.0720	0.0371	2.06	7.02E-06	0.0002
9	5	vr101	vr093	3.06E-06	5.00E-05	T CT GTCGAA	0.0541	0.0722	0.0371	2.07	6.87E-06	0.0001
10	5	vr101	vr092	3.17E-06	6.00E-05	T CT GTCGAAT	0.0540	0.0721	0.0371	2.07	6.78E-06	0.0001
11	5	vr101	vr091	5.44E-06	0.0001	T CT GTCGAATC	0.0537	0.0717	0.0371	2.04	9.59E-06	0.0002
12	5	vr101	vr090	7.05E-06	0.0001	T CT GTCGAATCC	0.0537	0.0718	0.0371	2.04	9.59E-06	0.0002
13	5	vr101	vr089	7.05E-06	0.0001	T CT GTCGAATCCA	0.0537	0.0718	0.0371	2.04	9.59E-06	0.0002
14	5	vr101	vr088	6.33E-06	0.0001	T CT GTCGAATCCAC	0.0537	0.0718	0.0371	2.04	9.59E-06	0.0002
15	5	vr101	vr087	7.47E-06	0.0001	T CT GTCGAATCCACC	0.0535	0.0713	0.0371	2.03	1.18E-05	0.0003
1	2	vr100	vr100	0.7000	0.9998	C	0.1820	0.1802	0.1841	0.97	0.7	1.0000
2	3	vr100	vr099	2.60E-07	2.00E-05	CT	0.0619	0.0788	0.0438	1.89	1.84E-05	0.0005
3	4	vr100	vr098	1.19E-06	2.00E-05	CT G	0.0604	0.0773	0.0426	1.91	1.67E-05	0.0004
4	4	vr100	vr097	3.40E-06	6.00E-05	CT GT	0.0599	0.0771	0.0427	1.90	2.05E-05	0.0005
5	4	vr100	vr096	4.33E-06	7.00E-05	CT GTC	0.0585	0.0756	0.0415	1.91	2.29E-05	0.0006
6	4	vr100	vr095	1.51E-06	4.00E-05	CT GTCG	0.0543	0.0723	0.0370	2.08	5.64E-06	0.0001
7	4	vr100	vr094	1.78E-06	4.00E-05	CT GTCGA	0.0543	0.0725	0.0371	2.08	5.72E-06	0.0001
8	4	vr100	vr093	2.02E-06	4.00E-05	CT GTCGAA	0.0544	0.0727	0.0371	2.08	5.59E-06	0.0001
9	4	vr100	vr092	2.13E-06	4.00E-05	CT GTCGAAT	0.0543	0.0726	0.0371	2.08	5.52E-06	0.0001
10	4	vr100	vr091	4.01E-06	7.00E-05	CT GTCGAATC	0.0540	0.0722	0.0371	2.06	7.83E-06	0.0002
11	4	vr100	vr090	5.73E-06	0.0001	CT GTCGAATCC	0.0540	0.0723	0.0371	2.06	7.83E-06	0.0002
12	4	vr100	vr089	5.73E-06	0.0001	CT GTCGAATCCA	0.0540	0.0723	0.0371	2.06	7.83E-06	0.0002
13	4	vr100	vr088	5.00E-06	8.00E-05	CT GTCGAATCCAC	0.0540	0.0723	0.0371	2.06	7.83E-06	0.0002
14	4	vr100	vr087	5.96E-06	0.0001	CT GTCGAATCCACC	0.0537	0.0718	0.0371	2.04	9.66E-06	0.0002
1	2	vr099	vr099	5.08E-05	0.0008	T	0.8750	0.8957	0.8528	1.53	5.08E-05	0.0013
2	3	vr099	vr098	1.62E-05	0.0003	T G	0.1240	0.1399	0.1061	1.37	0.00255	0.0549
3	4	vr099	vr097	1.14E-06	2.00E-05	TG T	0.0606	0.0784	0.0426	1.94	1.02E-05	0.0002
4	4	vr099	vr096	2.76E-06	5.00E-05	TG TC	0.0588	0.0760	0.0415	1.92	1.76E-05	0.0005
5	4	vr099	vr095	9.49E-07	2.00E-05	TG TC G	0.0546	0.0728	0.0370	2.10	4.40E-06	0.0001
6	4	vr099	vr094	1.10E-06	2.00E-05	TG TC GA	0.0546	0.0729	0.0371	2.10	4.36E-06	0.0001
7	4	vr099	vr093	1.28E-06	2.00E-05	TG TC GAA	0.0547	0.0731	0.0371	2.10	4.23E-06	0.0001
8	4	vr099	vr092	1.38E-06	4.00E-05	TG TC GAAT	0.0546	0.0730	0.0371	2.10	4.30E-06	0.0001
9	4	vr099	vr091	2.63E-06	5.00E-05	TG TC GAATC	0.0543	0.0726	0.0371	2.07	6.10E-06	0.0001
10	4	vr099	vr090	3.74E-06	7.00E-05	TG TC GAATCC	0.0543	0.0727	0.0370	2.07	5.94E-06	0.0001
11	4	vr099	vr089	3.74E-06	7.00E-05	TG TC GAATCCA	0.0543	0.0727	0.0370	2.07	5.94E-06	0.0001
12	4	vr099	vr088	3.26E-06	6.00E-05	TG TC GAATCCAC	0.0543	0.0727	0.0370	2.07	5.94E-06	0.0001
13	4	vr099	vr087	3.89E-06	7.00E-05	TG TC GAATCCACC	0.0540	0.0722	0.0371	2.06	7.33E-06	0.0002
1	2	vr098	vr098	0.620	0.998	G	0.2440	0.2417	0.2467	0.96	0.6200	1.0000
2	3	vr098	vr097	0.805	1.000	G T	0.1810	0.1810	0.1848	0.97	0.7300	1.0000
3	4	vr098	vr096	2.14E-06	4.00E-05	G TC	0.0593	0.0763	0.0420	1.93	1.50E-05	0.0003
4	4	vr098	vr095	7.20E-07	2.00E-05	G TC G	0.0552	0.0732	0.0376	2.10	3.71E-06	7.00E-05

Omnibus test (degree of freedom = H - 1)						Significant haplotype in the sliding window shown on the left						
Sliding window						Significant haplotype (*VIPR2* sense strand, 5'>3'; major allele in blue and minor allele in red)	Haplotype frequency in					
No. of SNPs per window	No. of common haplotypes (H; MHF>0.01)	First SNP	Last SNP	Asymptotic *P* value (P_a)	Empirical *P* value (P_{aemp})		Entire sample	Cases	Controls	Odds ratio	Asymptotic *P* value (P_a)	Empirical *P* value (P_{aemp})
5	4	vr098	vr094	8.66E-07	2.00E-05	G TC GA	0.0552	0.0733	0.0376	2.10	3.68E-06	7.00E-05
6	4	vr098	vr093	1.01E-06	2.00E-05	G TC GAA	0.0552	0.0735	0.0376	2.10	3.57E-06	7.00E-05
7	4	vr098	vr092	1.07E-06	2.00E-05	G TC GAAT	0.0551	0.0735	0.0376	2.11	3.53E-06	7.00E-05
8	4	vr098	vr091	1.50E-06	4.00E-05	G TC GAATC	0.0546	0.0730	0.0370	2.11	3.85E-06	8.00E-05
9	4	vr098	vr090	2.19E-06	5.00E-05	G TC GAATCC	0.0546	0.0732	0.0370	2.11	3.85E-06	8.00E-05
10	4	vr098	vr089	2.19E-06	5.00E-05	G TC GAATCCA	0.0546	0.0732	0.0370	2.11	3.85E-06	8.00E-05
11	4	vr098	vr088	1.88E-06	4.00E-05	G TC GAATCCAC	0.0546	0.0732	0.0370	2.11	3.85E-06	8.00E-05
12	4	vr098	vr087	2.26E-06	5.00E-05	G TC GAATCCACC	0.0543	0.0727	0.0371	2.09	4.77E-06	0.00011
1	2	vr097	vr097	0.597	0.9966	T	0.1870	0.1845	0.1901	0.95	0.5970	1.0000
2	3	vr097	vr096	5.36E-07	2.00E-05	TC	0.0593	0.0759	0.0417	1.93	1.50E-05	0.0003
3	4	vr097	vr095	4.64E-07	2.00E-05	TC G	0.0535	0.0710	0.0362	2.17	3.42E-06	7.00E-05
4	4	vr097	vr094	4.85E-07	2.00E-05	TC GA	0.0535	0.0712	0.0362	2.17	3.42E-06	7.00E-05
5	4	vr097	vr093	4.27E-07	2.00E-05	TC GAA	0.0550	0.0731	0.0373	2.13	3.14E-06	7.00E-05
6	4	vr097	vr092	5.08E-07	2.00E-05	TC GAAT	0.0550	0.0731	0.0374	2.11	3.38E-06	7.00E-05
7	4	vr097	vr091	7.20E-07	2.00E-05	TC GAATC	0.0544	0.0726	0.0368	2.12	3.64E-06	7.00E-05
8	4	vr097	vr090	1.07E-06	2.00E-05	TC GAATCC	0.0544	0.0727	0.0367	2.12	3.53E-06	7.00E-05
9	4	vr097	vr089	1.07E-06	2.00E-05	TC GAATCCA	0.0544	0.0727	0.0367	2.12	3.53E-06	7.00E-05
10	4	vr097	vr088	9.17E-07	2.00E-05	TC GAATCCAC	0.0544	0.0727	0.0368	2.12	3.52E-06	7.00E-05
11	4	vr097	vr087	1.09E-06	2.00E-05	TC GAATCCACC	0.0541	0.0722	0.0368	2.11	4.32E-06	0.0001
1	2	vr096	vr096	0.0001	0.0019	C	0.8730	0.8914	0.8516	1.48	0.0001	0.0036
2	3	vr096	vr095	0.0004	0.0050	CG	0.8050	0.8307	0.7870	1.37	0.0003	0.0063
3	3	vr096	vr094	0.0004	0.0051	CGA	0.8030	0.8315	0.7874	1.37	0.0003	0.0073
4	3	vr096	vr093	0.0005	0.0055	CGAA	0.8030	0.8350	0.7926	1.36	0.0004	0.0093
5	3	vr096	vr092	0.0005	0.0059	CGAAT	0.8030	0.8357	0.7929	1.36	0.0004	0.0099
6	3	vr096	vr091	0.0007	0.0082	CGAATC	0.8020	0.8356	0.7928	1.35	0.0005	0.0132
7	3	vr096	vr090	0.0012	0.0135	CGAATCC	0.8010	0.8358	0.7928	1.33	0.0008	0.0183
8	3	vr096	vr089	0.0012	0.0135	CGAATCCA	0.8010	0.8358	0.7928	1.33	0.0008	0.0183
9	3	vr096	vr088	0.0010	0.0112	CGAATCCAC	0.8010	0.8360	0.7927	1.34	0.0006	0.0151
10	3	vr096	vr087	0.0010	0.0113	CGAATCCACC	0.8010	0.8359	0.7930	1.34	0.0006	0.0151
1	2	vr095	vr095	0.0004	0.0048	G	0.8110	0.8316	0.7884	1.36	0.0004	0.0098
2	3	vr095	vr094	0.0005	0.0061	GA	0.8090	0.8314	0.7883	1.36	0.0004	0.0109
3	3	vr095	vr093	0.0005	0.0063	GAA	0.8090	0.8347	0.7934	1.35	0.0006	0.0145
4	3	vr095	vr092	0.0006	0.0067	GAAT	0.8080	0.8355	0.7942	1.34	0.0006	0.0151
5	3	vr095	vr091	0.0008	0.0095	GAATC	0.8070	0.8354	0.7942	1.33	0.0008	0.0197
6	3	vr095	vr090	0.0014	0.0156	GAATCC	0.8070	0.8356	0.7942	1.32	0.0012	0.0265
7	3	vr095	vr089	0.0014	0.0156	GAATCCA	0.8070	0.8356	0.7942	1.32	0.0012	0.0265
8	3	vr095	vr088	0.0013	0.0148	GAATCCAC	0.8070	0.8360	0.7940	1.33	0.0010	0.0233
9	3	vr095	vr087	0.0013	0.0149	GAATCCACC	0.8060	0.8359	0.7944	1.33	0.0010	0.0233
1	2	vr094	vr094	0.0002	0.0019	A	0.8780	0.8968	0.8576	1.49	0.0002	0.0036
2	2	vr094	vr093	0.0001	0.0017	AA	0.8730	0.8983	0.8583	1.48	0.0001	0.0033
3	2	vr094	vr092	0.0001	0.0018	AAT	0.8720	0.8987	0.8587	1.48	0.0001	0.0034
4	2	vr094	vr091	0.0002	0.0027	AATC	0.8710	0.8985	0.8586	1.46	0.0002	0.0050
5	2	vr094	vr090	0.0004	0.0045	AATCC	0.8700	0.8984	0.8586	1.44	0.0004	0.0090
6	2	vr094	vr089	0.0004	0.0045	AATCCA	0.8700	0.8984	0.8586	1.44	0.0004	0.0090
7	2	vr094	vr088	0.0004	0.0044	AATCCAC	0.8700	0.8983	0.8585	1.44	0.0004	0.0088
8	2	vr094	vr087	0.0004	0.0044	AATCCACC	0.8690	0.8983	0.8590	1.44	0.0004	0.0090
1	2	vr093	vr093	0.0001	0.0016	A	0.8750	0.8947	0.8540	1.49	0.0001	0.0031
2	2	vr093	vr092	0.0001	0.0015	AT	0.8730	0.8954	0.8557	1.49	0.0001	0.0029
3	2	vr093	vr091	0.0002	0.0022	ATC	0.8720	0.8953	0.8556	1.47	0.0002	0.0042
4	2	vr093	vr090	0.0003	0.0039	ATCC	0.8720	0.8985	0.8587	1.45	0.0003	0.0077
5	2	vr093	vr089	0.0003	0.0039	ATCCA	0.8720	0.8985	0.8587	1.45	0.0003	0.0077
6	2	vr093	vr088	0.0003	0.0038	ATCCAC	0.8710	0.8984	0.8586	1.45	0.0003	0.0075
7	2	vr093	vr087	0.0003	0.0038	ATCCACC	0.8700	0.8984	0.8590	1.44	0.0003	0.0076
1	2	vr092	vr092	0.0002	0.0023	T	0.8750	0.8936	0.8540	1.47	0.0002	0.0044
2	2	vr092	vr091	0.0003	0.0040	TC	0.8740	0.8939	0.8544	1.45	0.0003	0.0077
3	2	vr092	vr090	0.0004	0.0047	TCC	0.8720	0.8976	0.8578	1.44	0.0004	0.0094
4	2	vr092	vr089	0.0003	0.0040	TCCA	0.8720	0.8976	0.8577	1.45	0.0003	0.0079
5	2	vr092	vr088	0.0003	0.0039	TCCAC	0.8720	0.8975	0.8577	1.45	0.0003	0.0077
6	2	vr092	vr087	0.0003	0.0040	TCCACC	0.8710	0.8975	0.8581	1.45	0.0003	0.0077
1	2	vr091	vr091	0.0003	0.0037	C	0.8740	0.8925	0.8540	1.45	0.0003	0.0071
2	2	vr091	vr090	0.0004	0.0043	CC	0.8730	0.8971	0.8579	1.44	0.0004	0.0088
3	2	vr091	vr089	0.0003	0.0037	CCA	0.8730	0.8971	0.8578	1.45	0.0003	0.0073
4	2	vr091	vr088	0.0004	0.0044	CCAC	0.8720	0.8970	0.8577	1.44	0.0004	0.0088
5	2	vr091	vr087	0.0004	0.0044	CCACC	0.8710	0.8970	0.8582	1.44	0.0004	0.0090
1	2	vr090	vr090	0.0002	0.0024	C	0.8780	0.8963	0.8576	1.48	0.0002	0.0045
2	2	vr090	vr089	0.0002	0.0019	CA	0.8780	0.8963	0.8575	1.49	0.0002	0.0037
3	2	vr090	vr088	0.0002	0.0029	CAC	0.8760	0.8960	0.8583	1.47	0.0002	0.0054
4	2	vr090	vr087	0.0002	0.0031	CACC	0.8720	0.8956	0.8583	1.46	0.0002	0.0061

Omnibus test (degree of freedom = H - 1)						Significant haplotype in the sliding window shown on the left						
Sliding window							Haplotype frequency in					
No. of SNPs per window	No. of common haplotypes (H; MHF>0.01)	First SNP	Last SNP	Asymptotic *P* value (P_a)	Empirical *P* value (P_{aemp})	Significant haplotype (*VIPR2* sense strand, 5'>3'; major allele in blue and minor allele in red)	Entire sample	Cases	Controls	Odds ratio	Asymptotic *P* value (P_a)	Empirical *P* value (P_{aemp})
1	2	vr089	vr089	0.0002	0.0019	A	0.8780	0.8963	0.8570	1.49	0.0002	0.0037
2	2	vr089	vr088	0.0002	0.0029	AC	0.8760	0.8960	0.8583	1.47	0.0002	0.0054
3	2	vr089	vr087	0.0002	0.0031	ACC	0.8720	0.8956	0.8583	1.46	0.0002	0.0061
1	2	vr088	vr088	0.0004	0.0047	C	0.8770	0.8941	0.8576	1.45	0.0004	0.0096
2	2	vr088	vr087	0.0003	0.0037	CC	0.8720	0.8942	0.8578	1.45	0.0003	0.0072
1	2	vr087	vr087	0.0004	0.0044	C	0.8730	0.8909	0.8534	1.45	0.0004	0.0089
						**Protective haplotypes (odds ratio <1.00)**						
1	2	vr106	vr106	0.0004	0.0052	C	0.1070	0.0909	0.1257	0.68	0.0004	0.0108
2	2	vr106	vr105	0.0003	0.0040	CC	0.1000	0.0842	0.1199	0.66	0.0003	0.0067
3	2	vr106	vr104	0.0007	0.0078	CCG	0.0987	0.0831	0.1191	0.65	0.0002	0.0046
4	2	vr106	vr103	0.0015	0.0163	CCGT	0.0965	0.0833	0.1154	0.68	0.0009	0.0208
5	3	vr106	vr102	0.0046	0.0449	CCGTC	0.0953	0.0833	0.1132	0.69	0.0018	0.0406
1	2	vr105	vr105	0.0002	0.0026	C	0.1030	0.0856	0.1222	0.66	0.0002	0.0049
2	2	vr105	vr104	0.0006	0.0073	CG	0.1020	0.0845	0.1214	0.65	0.0001	0.0035
3	2	vr105	vr103	0.0014	0.0158	CGT	0.0985	0.0843	0.1167	0.68	0.0008	0.0184
4	3	vr105	vr102	0.0033	0.0330	CGTC	0.0973	0.0843	0.1146	0.70	0.0016	0.0359
1	2	vr104	vr104	0.0005	0.0055	G	0.1050	0.0893	0.1234	0.68	0.0005	0.0113
2	2	vr104	vr103	0.0016	0.0177	GT	0.0988	0.0847	0.1166	0.69	0.0009	0.0217
3	3	vr104	vr102	0.0033	0.0328	GTC	0.0976	0.0847	0.1145	0.70	0.0019	0.0425
1	2	vr102	vr102	0.0011	0.0130	C	0.1660	0.1471	0.1865	0.74	0.0011	0.0264
1	2	vr101	vr101	0.0013	0.0150	C	0.1030	0.0882	0.1198	0.70	0.0013	0.0302
1	2	vr100	vr100	0.7000	0.9998	C	0.1820	0.1802	0.1841	0.97	0.7000	1.0000
2	3	vr100	vr099	2.60E-07	2.00E-05	CG	0.1200	0.1019	0.1416	0.67	0.0002	0.0046
3	4	vr100	vr098	1.19E-06	2.00E-05	CGG	0.1200	0.1020	0.1417	0.67	0.0002	0.0046
4	4	vr100	vr097	3.40E-06	6.00E-05	CGGT	0.1200	0.1024	0.1418	0.67	0.0002	0.0046
5	4	vr100	vr096	4.33E-06	7.00E-05	CGGTT	0.1190	0.1021	0.1415	0.67	0.0002	0.0039
6	4	vr100	vr095	1.51E-06	4.00E-05	CGGTTC	0.1190	0.1028	0.1423	0.67	0.0002	0.0039
7	4	vr100	vr094	1.78E-06	4.00E-05	CGGTTCT	0.1190	0.1025	0.1419	0.67	0.0002	0.0038
8	4	vr100	vr093	2.02E-06	4.00E-05	CGGTTCTG	0.1180	0.1015	0.1409	0.67	0.0002	0.0044
9	4	vr100	vr092	2.13E-06	4.00E-05	CGGTTCTGC	0.1180	0.1016	0.1409	0.67	0.0002	0.0044
10	4	vr100	vr091	4.01E-06	7.00E-05	CGGTTCTGCG	0.1180	0.1017	0.1409	0.67	0.0002	0.0044
11	4	vr100	vr090	5.73E-06	0.00013	CGGTTCTGCGT	0.1180	0.1019	0.1409	0.67	0.0002	0.0044
12	4	vr100	vr089	5.73E-06	0.00013	CGGTTCTGCGTG	0.1180	0.1019	0.1409	0.67	0.0002	0.0044
13	4	vr100	vr088	5.00E-06	8.00E-05	CGGTTCTGCGTGT	0.1180	0.1019	0.1410	0.67	0.0002	0.0044
14	4	vr100	vr087	5.96E-06	0.0001	CGGTTCTGCGTGTT	0.1180	0.1019	0.1406	0.67	0.0002	0.0053
1	2	vr099	vr099	5.08E-05	0.0008	G	0.1250	0.1043	0.1472	0.66	5.08E-05	0.0013
2	3	vr099	vr098	1.62E-05	0.0003	GG	0.1200	0.1024	0.1421	0.67	0.0002	0.0043
3	4	vr099	vr097	1.14E-06	2.00E-05	GGT	0.1200	0.1026	0.1422	0.67	0.0002	0.0043
4	4	vr099	vr096	2.76E-06	5.00E-05	GGTT	0.1200	0.1024	0.1418	0.67	0.0002	0.0036
5	4	vr099	vr095	9.49E-07	2.00E-05	GGTTC	0.1200	0.1031	0.1428	0.67	0.0002	0.0036
6	4	vr099	vr094	1.10E-06	2.00E-05	GGTTCT	0.1190	0.1028	0.1423	0.67	0.0002	0.0036
7	4	vr099	vr093	1.28E-06	2.00E-05	GGTTCTG	0.1180	0.1019	0.1413	0.67	0.0002	0.0042
8	4	vr099	vr092	1.38E-06	4.00E-05	GGTTCTGC	0.1180	0.1019	0.1414	0.67	0.0002	0.0042
9	4	vr099	vr091	2.63E-06	5.00E-05	GGTTCTGCG	0.1180	0.1020	0.1414	0.67	0.0002	0.0042
10	4	vr099	vr090	3.74E-06	7.00E-05	GGTTCTGCGT	0.1180	0.1022	0.1414	0.67	0.0002	0.0042
11	4	vr099	vr089	3.74E-06	7.00E-05	GGTTCTGCGTG	0.1180	0.1022	0.1414	0.67	0.0002	0.0042
12	4	vr099	vr088	3.26E-06	6.00E-05	GGTTCTGCGTGT	0.1180	0.1022	0.1415	0.67	0.0002	0.0042
13	4	vr099	vr087	3.89E-06	7.00E-05	GGTTCTGCGTGTT	0.1180	0.1022	0.1410	0.67	0.0002	0.0051
1	2	vr098	vr098	0.6200	0.9981	G	0.2440	0.2417	0.2467	0.96	0.6200	1.0000
2	3	vr098	vr097	0.8050	1.0000	GT	0.1810	0.1810	0.1848	0.97	0.7300	1.0000
3	4	vr098	vr096	2.14E-06	4.00E-05	GTT	0.1220	0.1047	0.1428	0.68	0.0003	0.0068
4	4	vr098	vr095	7.20E-07	2.00E-05	GTTC	0.1210	0.1044	0.1437	0.67	0.0002	0.0042
5	4	vr098	vr094	8.66E-07	2.00E-05	GTTCT	0.1210	0.1037	0.1433	0.67	0.0002	0.0036
6	4	vr098	vr093	1.01E-06	2.00E-05	GTTCTG	0.1190	0.1028	0.1423	0.67	0.0002	0.0042
7	4	vr098	vr092	1.07E-06	2.00E-05	GTTCTGC	0.1190	0.1023	0.1423	0.67	0.0001	0.0036
8	4	vr098	vr091	1.50E-06	4.00E-05	GTTCTGCG	0.1190	0.1025	0.1424	0.67	0.0001	0.0036
9	4	vr098	vr090	2.19E-06	5.00E-05	GTTCTGCGT	0.1190	0.1026	0.1424	0.67	0.0001	0.0036
10	4	vr098	vr089	2.19E-06	5.00E-05	GTTCTGCGTG	0.1190	0.1026	0.1424	0.67	0.0001	0.0036
11	4	vr098	vr088	1.88E-06	4.00E-05	GTTCTGCGTGT	0.1190	0.1026	0.1425	0.67	0.0001	0.0036
12	4	vr098	vr087	2.26E-06	5.00E-05	GTTCTGCGTGTT	0.1190	0.1027	0.1421	0.67	0.0002	0.0042
1	2	vr097	vr097	0.5970	0.9966	T	0.1870	0.1845	0.1901	0.95	0.5970	1.0000
2	3	vr097	vr096	5.36E-07	2.00E-05	TT	0.1270	0.1086	0.1484	0.68	0.0001	0.0036
3	4	vr097	vr095	4.64E-07	2.00E-05	TTC	0.1210	0.1048	0.1436	0.68	0.0002	0.0054
4	4	vr097	vr094	4.85E-07	2.00E-05	TTCT	0.1210	0.1035	0.1433	0.67	0.0002	0.0036
5	4	vr097	vr093	4.27E-07	2.00E-05	TTCTG	0.1190	0.1024	0.1422	0.67	0.0002	0.0042

Omnibus test (degree of freedom = H - 1)						Significant haplotype in the sliding window shown on the left						
Sliding window						Significant haplotype (*VIPR2* sense strand, 5'>3'; major allele in blue and minor allele in red)	Haplotype frequency in					
No. of SNPs per window	No. of common haplotypes (H; MHF>0.01)	First SNP	Last SNP	Asymptotic *P* value (P_a)	Empirical *P* value (P_{aemp})		Entire sample	Cases	Controls	Odds ratio	Asymptotic *P* value (P_a)	Empirical *P* value (P_{aemp})
6	4	vr097	vr092	5.08E-07	2.00E-05	TTCTGC	0.1190	0.1020	0.1423	0.67	0.0001	0.0036
7	4	vr097	vr091	7.20E-07	2.00E-05	TTCTGCG	0.1190	0.1021	0.1424	0.67	0.0001	0.0036
8	4	vr097	vr090	1.07E-06	2.00E-05	TTCTGCGT	0.1190	0.1022	0.1424	0.67	0.0001	0.0036
9	4	vr097	vr089	1.07E-06	2.00E-05	TTCTGCGTG	0.1190	0.1022	0.1424	0.67	0.0001	0.0036
10	4	vr097	vr088	9.17E-07	2.00E-05	TTCTGCGTGT	0.1190	0.1022	0.1425	0.67	0.0001	0.0036
11	4	vr097	vr087	1.09E-06	2.00E-05	TTCTGCGTGTT	0.1190	0.1023	0.1420	0.67	0.0002	0.0042
1	2	vr096	vr096	0.0001	0.0019	T	0.1270	0.1086	0.1484	0.68	0.0001	0.0036
2	3	vr096	vr095	0.0004	0.0050	TC	0.1210	0.1042	0.1428	0.68	0.0002	0.0054
3	3	vr096	vr094	0.0004	0.0051	TCT	0.1210	0.1029	0.1424	0.67	0.0002	0.0036
4	3	vr096	vr093	0.0005	0.0055	TCTG	0.1190	0.1023	0.1421	0.67	0.0002	0.0042
5	3	vr096	vr092	0.0005	0.0059	TCTGC	0.1190	0.1020	0.1423	0.67	0.0001	0.0036
6	3	vr096	vr091	0.0007	0.0082	TCTGCG	0.1190	0.1021	0.1423	0.67	0.0001	0.0036
7	3	vr096	vr090	0.0012	0.0135	TCTGCGT	0.1190	0.1022	0.1423	0.67	0.0001	0.0036
8	3	vr096	vr089	0.0012	0.0135	TCTGCGTG	0.1190	0.1022	0.1423	0.67	0.0001	0.0036
9	3	vr096	vr088	0.0010	0.0112	TCTGCGTGT	0.1190	0.1022	0.1424	0.67	0.0001	0.0036
10	3	vr096	vr087	0.0010	0.0113	TCTGCGTGTT	0.1190	0.1023	0.1420	0.67	0.0002	0.0042
1	2	vr095	vr095	0.0004	0.0048	C	0.1890	0.1684	0.2116	0.74	0.0004	0.0098
2	3	vr095	vr094	0.0005	0.0061	CT	0.1210	0.1022	0.1419	0.67	0.0001	0.0031
3	3	vr095	vr093	0.0005	0.0063	CTG	0.1200	0.1017	0.1417	0.67	0.0001	0.0035
4	3	vr095	vr092	0.0006	0.0067	CTGC	0.1190	0.1013	0.1413	0.67	0.0001	0.0036
5	3	vr095	vr091	0.0008	0.0095	CTGCG	0.1190	0.1015	0.1414	0.67	0.0001	0.0036
6	3	vr095	vr090	0.0014	0.0156	CTGCGT	0.1190	0.1016	0.1414	0.67	0.0001	0.0036
7	3	vr095	vr089	0.0014	0.0156	CTGCGTG	0.1190	0.1016	0.1414	0.67	0.0001	0.0036
8	3	vr095	vr088	0.0013	0.0148	CTGCGTGT	0.1190	0.1017	0.1415	0.67	0.0001	0.0036
9	3	vr095	vr087	0.0013	0.0149	CTGCGTGTT	0.1190	0.1017	0.1410	0.67	0.0002	0.0042
1	2	vr094	vr094	0.0002	0.0019	T	0.1220	0.1032	0.1424	0.67	0.0002	0.0036
2	2	vr094	vr093	0.0001	0.0017	TG	0.1200	0.1017	0.1417	0.67	0.0001	0.0035
3	2	vr094	vr092	0.0001	0.0018	TGC	0.1190	0.1013	0.1413	0.67	0.0001	0.0035
4	2	vr094	vr091	0.0002	0.0027	TGCG	0.1190	0.1015	0.1414	0.67	0.0001	0.0036
5	2	vr094	vr090	0.0004	0.0045	TGCGT	0.1190	0.1016	0.1414	0.67	0.0001	0.0036
6	2	vr094	vr089	0.0004	0.0045	TGCGTG	0.1190	0.1016	0.1414	0.67	0.0001	0.0036
7	2	vr094	vr088	0.0004	0.0044	TGCGTGT	0.1190	0.1017	0.1415	0.67	0.0001	0.0036
8	2	vr094	vr087	0.0004	0.0044	TGCGTGTT	0.1190	0.1017	0.1410	0.67	0.0002	0.0042
1	2	vr093	vr093	0.0001	0.0016	G	0.1250	0.1053	0.1460	0.67	0.0001	0.0031
2	2	vr093	vr092	0.0001	0.0015	GC	0.1230	0.1046	0.1443	0.68	0.0002	0.0048
3	2	vr093	vr091	0.0002	0.0022	GCG	0.1230	0.1047	0.1444	0.68	0.0002	0.0048
4	2	vr093	vr090	0.0003	0.0039	GCGT	0.1190	0.1015	0.1413	0.67	0.0001	0.0035
5	2	vr093	vr089	0.0003	0.0039	GCGTG	0.1190	0.1015	0.1413	0.67	0.0001	0.0035
6	2	vr093	vr088	0.0003	0.0038	GCGTGT	0.1190	0.1016	0.1414	0.67	0.0001	0.0036
7	2	vr093	vr087	0.0003	0.0038	GCGTGTT	0.1190	0.1016	0.1410	0.67	0.0002	0.0042
1	2	vr092	vr092	0.0002	0.0023	C	0.1250	0.1064	0.1460	0.68	0.0002	0.0044
2	2	vr092	vr091	0.0003	0.0040	CG	0.1250	0.1061	0.1456	0.68	0.0002	0.0041
3	2	vr092	vr090	0.0004	0.0047	CGT	0.1200	0.1024	0.1422	0.67	0.0001	0.0031
4	2	vr092	vr089	0.0003	0.0040	CGTG	0.1200	0.1024	0.1423	0.67	0.0001	0.0031
5	2	vr092	vr088	0.0003	0.0039	CGTGT	0.1200	0.1025	0.1423	0.67	0.0001	0.0031
6	2	vr092	vr087	0.0003	0.0040	CGTGTT	0.1200	0.1025	0.1419	0.67	0.0002	0.0036
1	2	vr091	vr091	0.0003	0.0037	G	0.1260	0.1075	0.1460	0.69	0.0003	0.0071
2	2	vr091	vr090	0.0004	0.0043	GT	0.1210	0.1029	0.1421	0.67	0.0002	0.0036
3	2	vr091	vr089	0.0003	0.0037	GTG	0.1210	0.1029	0.1422	0.67	0.0002	0.0036
4	2	vr091	vr088	0.0004	0.0044	GTGT	0.1210	0.1030	0.1423	0.67	0.0002	0.0036
5	2	vr091	vr087	0.0004	0.0044	GTGTT	0.1200	0.1030	0.1418	0.67	0.0002	0.0044
1	2	vr090	vr090	0.0002	0.0024	T	0.1220	0.1037	0.1424	0.68	0.0002	0.0045
2	2	vr090	vr089	0.0002	0.0019	TG	0.1220	0.1037	0.1425	0.68	0.0002	0.0045
3	2	vr090	vr088	0.0002	0.0029	TGT	0.1210	0.1040	0.1417	0.68	0.0003	0.0069
4	2	vr090	vr087	0.0002	0.0031	TGTT	0.1210	0.1044	0.1417	0.69	0.0003	0.0083
1	2	vr089	vr089	0.0002	0.0019	G	0.1220	0.1037	0.1430	0.67	0.0002	0.0037
2	2	vr089	vr088	0.0002	0.0029	GT	0.1210	0.1040	0.1417	0.68	0.0003	0.0069
3	2	vr089	vr087	0.0002	0.0031	GTT	0.1210	0.1044	0.1417	0.69	0.0003	0.0083
1	2	vr088	vr088	0.0004	0.0047	T	0.1230	0.1059	0.1424	0.69	0.0004	0.0096
2	2	vr088	vr087	0.0003	0.0037	TT	0.1220	0.1058	0.1422	0.69	0.0005	0.0114
1	2	vr087	vr087	0.0004	0.0044	T	0.1270	0.1091	0.1466	0.69	0.0004	0.0089

* The sliding window is shifted one SNP at a time towards the 3' end of the *VIPR2* gene. A small number of sliding windows or haplotypes with P_{aemp} >0.05 (i.e. not significant) are included to illustrate the patterns, and shown in grey for easy distinction. The alleles are coded based on the sense strand of the *VIPR2* gene.

The most significant haplotype window is the 2-SNP window of vr100-vr099 (P_a = 2.60×10^{-7} shown above in **red and boldface**).

The most significant haplotype is the 8-SNP haplotype TTACGT **CT** of vr106-vr099 (P_a = 1.03×10^{-6} shown above in **red and boldface**).

Supplementary Table 7. Summary of single-marker analysis of imputed genotypes by SNPTEST based on dosage: significant SNPs*

SNP	Sequential numbering (among 368 SNPs)	Position on Chr 7 (hg19)	Alleles minor/major	Minor allele frequency in			Odds ratio	Asymptotic *P* value	Rank	P_{cor}	$-\log_{10}(P_{cor})$
				All samples	Cases	Controls					
**Significant SNPs (n=24) with high-risk minor alleles (odds ratio > 1.00)**											
rs77572211	364	158,945,743	T/C	0.0931	0.1143	0.0695	1.73	2.37E-08	7	1.25E-06	5.905
rs74699763	350	158,937,357	C/G	0.1089	0.1310	0.0844	1.63	9.95E-09	4	9.15E-07	6.038
rs4518608	349	158,934,935	C/T	0.1031	0.1252	0.0785	1.68	6.01E-09	3	7.37E-07	6.132
rs77003675	347	158,934,102	A/G	0.1048	0.1261	0.0811	1.63	1.86E-08	6	1.14E-06	5.943
rs76649997	346	158,933,824	G/A	0.1070	0.1280	0.0835	1.61	2.88E-08	8	1.32E-06	5.878
rs75522535	342	158,932,199	C/G	0.0969	0.1205	0.0706	1.80	2.46E-10	1	9.05E-08	7.043
rs79254483	341	158,931,850	T/C	0.1033	0.1256	0.0785	1.69	2.54E-09	2	4.67E-07	6.330
rs188030062	334	158,930,035	G/T	0.0203	0.0285	0.0111	2.60	1.81E-08	5	1.33E-06	5.875
rs75247079	330	158,929,959	G/T	0.1088	0.1290	0.0863	1.57	1.32E-07	9	5.40E-06	5.268
7:158925730	320	158,925,730	A/G	0.0963	0.1146	0.0759	1.58	1.40E-07	10	5.15E-06	5.288
rs74790021	307	158,920,661	T/C	0.0784	0.0935	0.0616	1.57	4.85E-05	19	9.39E-04	3.027
rs6950857	282	158,910,937	A/G	0.1060	0.1192	0.0913	1.35	0.0015	99	0.0055	2.261
rs373808146	227	158,894,371	A/G	0.0791	0.0961	0.0601	1.66	9.09E-06	16	2.09E-04	3.680
rs76583299	224	158,893,378	T/C	0.0766	0.0944	0.0569	1.73	3.98E-06	13	1.13E-04	3.948
rs79725155	212	158,886,957	T/C	0.0765	0.0942	0.0569	1.72	4.27E-06	14	1.12E-04	3.950
rs143679615	191	158,880,540	G/A	0.0657	0.0820	0.0474	1.79	7.03E-07	11	2.35E-05	4.629
rs185588523	190	158,880,539	G/C	0.0764	0.0941	0.0567	1.73	3.53E-06	12	1.08E-04	3.966
rs10257329	189	158,880,495	G/C	0.0805	0.0976	0.0615	1.65	1.07E-05	17	2.32E-04	3.635
rs3812305	177	158,875,666	T/C	0.0841	0.1010	0.0654	1.61	1.69E-05	18	3.46E-04	3.462
rs78522600	156	158,870,187	GT/G	0.0845	0.1028	0.0641	1.67	5.10E-06	15	1.25E-04	3.903
rs6950938	77	158,828,355	T/C	0.1666	0.1796	0.1521	1.22	0.0059	138	0.0158	1.800
rs12113506	58	158,818,164	T/C	0.0507	0.0633	0.0366	1.78	1.16E-04	21	0.0020	2.692
rs186428185	34	158,798,680	G/T	0.1330	0.1432	0.1217	1.21	0.0160	183	0.0322	1.492
vr037 (rs76749764)	26	158,788,690	T/C	0.1136	0.1236	0.1026	1.23	0.0264	197	0.0494	1.306
**Significant (n=173) SNPs with protective minor alleles (odds ratio < 1.00)**											
rs396179	368	158,951,983	C/T	0.1058	0.0947	0.1180	0.78	0.0037	110	0.0124	1.907
rs36092265	366	158,946,357	A/C	0.0991	0.0889	0.1105	0.79	0.0092	168	0.0202	1.694
rs35837417	365	158,946,211	C/T	0.1018	0.0917	0.1131	0.79	0.0094	169	0.0206	1.687
rs35912991	363	158,945,539	T/C	0.0985	0.0887	0.1095	0.79	0.0122	173	0.0258	1.588
rs759001	362	158,943,746	G/T	0.1712	0.1589	0.1848	0.83	0.0071	150	0.0175	1.758
rs751220	361	158,942,971	G/A	0.1789	0.1653	0.1940	0.82	0.0040	113	0.0131	1.883
rs35029789	360	158,942,650	G/A	0.1023	0.0932	0.1125	0.81	0.0184	191	0.0354	1.450
rs34863862	359	158,942,433	T/C	0.1001	0.0906	0.1106	0.80	0.0152	178	0.0314	1.504
rs34301429	358	158,942,397	T/G	0.1136	0.1015	0.1270	0.78	0.0027	105	0.0095	2.022
rs35421629	357	158,940,806	C/T	0.0983	0.0887	0.1089	0.80	0.0145	177	0.0302	1.520
rs6967089	356	158,940,578	G/A	0.1515	0.1411	0.1631	0.84	0.0178	190	0.0344	1.463
rs72245971	355	158,939,772	A/ATATCTGTGATCTGCCT	0.1462	0.1359	0.1577	0.84	0.0174	189	0.0339	1.469
rs28385319	354	158,939,764	C/G	0.1462	0.1359	0.1577	0.84	0.0174	188	0.0341	1.467
rs28487949	353	158,939,761	A/T	0.1462	0.1359	0.1577	0.84	0.0174	187	0.0343	1.465
rs4909115	352	158,938,201	T/C	0.1042	0.0948	0.1147	0.81	0.0157	181	0.0319	1.497
rs3828968	351	158,937,954	A/G	0.1029	0.0929	0.1140	0.80	0.0113	172	0.0242	1.616
rs3812314	345	158,933,239	C/A	0.1035	0.0913	0.1171	0.76	0.0038	111	0.0127	1.896
rs202180257	344	158,932,763	T/TTTTTTG	0.0966	0.0855	0.1088	0.77	0.0071	151	0.0174	1.761
rs3812313	343	158,932,459	A/G	0.0965	0.0855	0.1088	0.77	0.0072	152	0.0174	1.759
rs34934389	340	158,931,735	A/G	0.1026	0.0911	0.1155	0.77	0.0056	132	0.0156	1.808
rs35595971	339	158,931,067	C/T	0.1034	0.0912	0.1168	0.76	0.0040	114	0.0130	1.884
rs3812312	338	158,930,775	A/G	0.0964	0.0854	0.1087	0.77	0.0073	157	0.0171	1.766
7:158929978	333	158,929,978	CATAAAGAA/C	0.0965	0.0855	0.1088	0.77	0.0072	155	0.0171	1.766
7:158929976	332	158,929,976	A/T	0.0965	0.0855	0.1088	0.77	0.0072	154	0.0173	1.763
rs202247208	331	158,929,974	AAAG/A	0.0965	0.0855	0.1088	0.77	0.0072	153	0.0174	1.760
rs34772671	329	158,929,779	A/G	0.0975	0.0858	0.1105	0.76	0.0047	120	0.0145	1.839
rs440328	328	158,929,243	C/A	0.1600	0.1456	0.1762	0.80	0.0023	102	0.0085	2.073
rs59845329	327	158,929,002	C/CG	0.0931	0.0805	0.1070	0.73	7.41E-04	79	0.0035	2.462
rs6459928	326	158,928,569	T/C	0.1444	0.1325	0.1576	0.82	0.0106	171	0.0228	1.642
rs435077	325	158,928,427	T/C	0.1947	0.1766	0.2148	0.78	3.57E-04	44	0.0030	2.525
rs12234576	324	158,928,375	A/G	0.1468	0.1352	0.1597	0.82	0.0158	182	0.0320	1.495
rs429893	323	158,927,015	A/G	0.2069	0.1868	0.2293	0.77	1.18E-04	22	0.0020	2.704
rs55732438	319	158,923,783	G/A	0.1028	0.0881	0.1191	0.71	0.0014	94	0.0055	2.257
vr152 (rs56382838)	318	158,923,337	G/A	0.1045	0.0878	0.1231	0.69	3.31E-04	38	0.0032	2.494
vr151 (rs9691129)	312	158,921,796	C/G	0.1038	0.0881	0.1213	0.70	6.62E-04	74	0.0033	2.483
vr150 (rs3828963)	308	158,920,678	T/A	0.1013	0.0850	0.1193	0.69	4.66E-04	58	0.0030	2.529
rs3812307	306	158,920,606	A/G	0.1006	0.0847	0.1184	0.69	5.80E-04	71	0.0030	2.522
rs3793241	304	158,919,442	C/T	0.1869	0.1728	0.2025	0.82	0.0163	184	0.0326	1.487
vr149 (rs67373805)	303	158,918,770	C/T	0.1062	0.0899	0.1243	0.70	5.54E-04	67	0.0030	2.517
rs73169267	299	158,917,416	T/C	0.1021	0.0865	0.1195	0.70	8.47E-04	85	0.0037	2.436
rs6459927	298	158,917,248	A/T	0.1785	0.1658	0.1926	0.83	0.0258	196	0.0484	1.315
rs58611395	297	158,915,899	A/G	0.1002	0.0845	0.1176	0.69	6.70E-04	75	0.0033	2.483
vr148 (rs59630301)	295	158,915,593	G/A	0.1023	0.0862	0.1201	0.69	5.56E-04	68	0.0030	2.522
rs72410189	294	158,915,395	G/GCAGATGTAA	0.0992	0.0842	0.1160	0.70	0.0011	89	0.0044	2.355
rs55807738	293	158,915,306	G/A	0.1689	0.1536	0.1858	0.80	0.0071	149	0.0176	1.756
vr147 (rs56157635)	292	158,915,211	T/C	0.1043	0.0875	0.1229	0.68	3.28E-04	37	0.0033	2.486
vr146 (rs17837881)	291	158,915,082	T/C	0.1007	0.0844	0.1188	0.68	4.45E-04	54	0.0030	2.518
vr145 (rs56376138)	290	158,914,887	G/A	0.1072	0.0896	0.1268	0.68	2.03E-04	25	0.0030	2.524

SNP	Sequential numbering (among 368 SNPs)	Position on Chr 7 (hg19)	Alleles minor/major	Minor allele frequency in All samples	Cases	Controls	Odds ratio	Asymptotic *P* value	Rank	P_{cor}	$-\log_{10}(P_{cor})$
vr144 (rs17837880)	289	158,914,795	T/C	0.1016	0.0850	0.1201	0.68	3.42E-04	42	0.0030	2.523
rs6459925	288	158,914,209	A/G	0.1890	0.1752	0.2044	0.83	0.0154	179	0.0317	1.500
vr142 (rs73169264)	285	158,912,891	A/G	0.1016	0.0856	0.1193	0.69	6.04E-04	73	0.0030	2.516
rs7787045	284	158,912,576	C/T	0.1908	0.1762	0.2071	0.82	0.0126	174	0.0267	1.574
vr141 (rs3793237)	283	158,912,033	T/C	0.1008	0.0848	0.1187	0.69	5.69E-04	70	0.0030	2.525
rs149551940	275	158,908,640	GGT/G	0.1691	0.1537	0.1862	0.79	0.0078	162	0.0177	1.751
vr139 (rs55776245)	274	158,908,527	T/C	0.1004	0.0842	0.1185	0.68	5.24E-04	64	0.0030	2.521
rs6973238	273	158,908,296	A/G	0.1692	0.1538	0.1864	0.79	0.0076	160	0.0175	1.757
rs6974127	272	158,908,079	T/C	0.1693	0.1538	0.1866	0.79	0.0073	158	0.0171	1.767
vr138 (rs6459924)	271	158,908,074	C/T	0.1755	0.1593	0.1936	0.79	0.0058	134	0.0159	1.797
rs17837879	270	158,907,664	T/G	0.1694	0.1538	0.1867	0.79	0.0073	156	0.0172	1.764
rs138810767	269	158,907,492	TAGAA/T	0.1670	0.1516	0.1841	0.79	0.0071	148	0.0176	1.755
rs73730165	268	158,906,055	C/T	0.1701	0.1543	0.1876	0.79	0.0066	145	0.0167	1.776
rs111591686	266	158,905,966	T/C	0.1660	0.1519	0.1817	0.81	0.0129	175	0.0272	1.565
rs56366955	265	158,905,777	T/C	0.1751	0.1588	0.1932	0.79	0.0054	129	0.0155	1.809
rs34740600	263	158,904,370	CA/C	0.1763	0.1611	0.1932	0.80	0.0083	163	0.0186	1.730
rs6952092	262	158,904,356	A/G	0.1703	0.1544	0.1879	0.79	0.0063	143	0.0162	1.791
rs6976646	261	158,904,302	C/T	0.1764	0.1597	0.1949	0.78	0.0048	121	0.0147	1.831
rs6972331	260	158,904,244	G/A	0.1764	0.1597	0.1949	0.78	0.0048	122	0.0146	1.835
rs6459923	259	158,903,371	G/C	0.1697	0.1539	0.1872	0.79	0.0067	147	0.0167	1.777
rs17837875	258	158,903,083	C/T	0.1064	0.0893	0.1255	0.68	3.01E-04	34	0.0033	2.487
rs3750072	257	158,902,741	G/A	0.1697	0.1539	0.1872	0.79	0.0066	146	0.0167	1.777
vr137 (rs3793232)	255	158,902,425	C/T	0.1760	0.1597	0.1941	0.79	0.0058	136	0.0158	1.802
vr136 (rs3793231)	254	158,902,148	A/G	0.1760	0.1592	0.1948	0.78	0.0043	116	0.0137	1.862
vr135 (rs6459922)	253	158,901,893	T/C	0.1760	0.1591	0.1947	0.78	0.0044	118	0.0137	1.863
vr134 (rs55708631)	252	158,901,643	T/C	0.1003	0.0836	0.1190	0.68	3.54E-04	43	0.0030	2.519
rs3793230	251	158,900,939	A/G	0.1707	0.1551	0.1880	0.79	0.0074	159	0.0172	1.765
rs3793229	250	158,900,731	T/C	0.1763	0.1598	0.1947	0.79	0.0053	127	0.0153	1.815
vr133 (rs3828962)	248	158,900,606	C/T	0.0984	0.0836	0.1149	0.70	0.0014	95	0.0055	2.258
rs2864943	247	158,899,811	C/T	0.1765	0.1601	0.1948	0.79	0.0056	131	0.0156	1.806
vr132 (rs55950988)	245	158,898,659	T/C	0.1065	0.0892	0.1257	0.68	2.81E-04	28	0.0037	2.433
rs2864944	244	158,898,286	A/T	0.1706	0.1562	0.1867	0.81	0.0132	176	0.0276	1.558
rs55789539	239	158,897,238	C/T	0.2008	0.1822	0.2215	0.78	0.0012	90	0.0049	2.308
vr131 (rs2270314)	238	158,896,624	G/C	0.1040	0.0865	0.1234	0.67	2.31E-04	27	0.0032	2.502
rs2270313	237	158,896,517	A/G	0.1563	0.1407	0.1736	0.78	0.0050	124	0.0149	1.826
vr130 (rs73169251)	235	158,896,036	G/C	0.1030	0.0860	0.1218	0.68	3.69E-04	45	0.0030	2.520
vr129 (rs73169250)	234	158,895,803	A/G	0.1009	0.0839	0.1197	0.67	2.98E-04	33	0.0033	2.478
vr128 (rs73169249)	233	158,895,796	A/C	0.1008	0.0839	0.1197	0.67	2.98E-04	32	0.0034	2.465
rs55924063	232	158,894,878	G/A	0.1028	0.0864	0.1210	0.69	4.16E-04	50	0.0031	2.514
rs56293137	230	158,894,710	G/A	0.1070	0.0903	0.1256	0.69	4.69E-04	60	0.0029	2.541
rs6459921	229	158,894,467	T/C	0.1863	0.1698	0.2046	0.80	0.0061	139	0.0161	1.793
rs12666392	226	158,893,700	C/T	0.1805	0.1636	0.1993	0.79	0.0050	125	0.0148	1.829
rs55842173	225	158,893,533	C/T	0.1024	0.0861	0.1206	0.69	5.58E-04	69	0.0030	2.526
vr127 (rs72621132)	223	158,893,193	C/T	0.1802	0.1636	0.1988	0.79	0.0057	133	0.0158	1.802
vr126 (rs73169248)	219	158,891,742	G/T	0.1029	0.0862	0.1216	0.68	4.68E-04	59	0.0029	2.535
rs3793228	218	158,891,506	G/A	0.1753	0.1590	0.1934	0.79	0.0062	140	0.0162	1.790
rs73169246	217	158,890,593	T/C	0.1010	0.0850	0.1187	0.69	7.16E-04	77	0.0034	2.466
rs3838701	215	158,888,507	C/CT	0.1806	0.1633	0.1999	0.78	0.0040	112	0.0130	1.885
vr125 (rs73169245)	214	158,887,358	G/C	0.1076	0.0904	0.1267	0.69	4.40E-04	53	0.0031	2.515
rs73169244	213	158,887,293	C/T	0.1015	0.0856	0.1191	0.69	7.95E-04	82	0.0036	2.447
rs147157741	211	158,886,417	G/C	0.1076	0.0916	0.1254	0.70	8.95E-04	86	0.0038	2.417
vr124 (rs3793227)	210	158,885,928	G/A	0.1066	0.0901	0.1249	0.69	7.32E-04	78	0.0035	2.462
rs7797488	209	158,884,855	C/A	0.1784	0.1617	0.1970	0.79	0.0056	130	0.0157	1.803
rs3793226	208	158,884,749	T/C	0.1029	0.0883	0.1191	0.72	0.0024	103	0.0086	2.064
vr123 (rs73169239)	207	158,884,305	A/C	0.1037	0.0877	0.1214	0.70	8.42E-04	84	0.0037	2.433
vr122 (rs73169238)	204	158,883,818	A/G	0.1033	0.0863	0.1223	0.68	3.76E-04	46	0.0030	2.521
rs370613428	201	158,883,595	C/A	0.0994	0.0860	0.1144	0.73	0.0028	108	0.0096	2.016
rs14912955	199	158,882,743	A/G	0.1066	0.0918	0.1232	0.72	0.0015	98	0.0055	2.258
rs73169236	198	158,882,706	T/C	0.1734	0.1573	0.1913	0.79	0.0063	141	0.0163	1.787
rs67464981	197	158,882,703	C/T	0.1791	0.1625	0.1976	0.79	0.0058	135	0.0158	1.800
rs66942973	196	158,882,409	A/G	0.1791	0.1625	0.1975	0.79	0.0059	137	0.0159	1.799
vr121 (rs73169234)	195	158,882,366	T/C	0.1030	0.0863	0.1217	0.68	4.66E-04	57	0.0030	2.522
vr120 (rs73169233)	194	158,882,201	T/C	0.1031	0.0863	0.1217	0.68	4.65E-04	56	0.0031	2.515
vr119 (rs55683317)	187	158,878,599	T/C	0.1015	0.0863	0.1185	0.70	0.0014	91	0.0055	2.262
rs113222672	186	158,878,407	G/GT	0.1719	0.1578	0.1875	0.81	0.0156	180	0.0319	1.497
vr118 (rs3793224)	182	158,876,573	A/G	0.1065	0.0890	0.1259	0.68	2.84E-04	30	0.0035	2.458
vr117 (rs56260213)	179	158,875,921	T/C	0.1065	0.0895	0.1254	0.69	4.18E-04	51	0.0030	2.520
vr116 (rs56023468)	174	158,875,329	C/T	0.1070	0.0895	0.1265	0.68	2.85E-04	31	0.0034	2.471
rs113531517	169	158,874,950	T/C	0.1844	0.1659	0.2050	0.77	0.0024	104	0.0086	2.063
rs370397262	166	158,873,427	C/A	0.1066	0.0895	0.1257	0.68	3.88E-04	47	0.0030	2.517
rs12668528	162	158,871,867	C/T	0.1842	0.1660	0.2045	0.77	0.0028	107	0.0097	2.013
rs12668638	161	158,871,812	T/C	0.1842	0.1660	0.2045	0.77	0.0028	106	0.0098	2.009
rs3812304	160	158,871,744	A/G	0.1711	0.1548	0.1894	0.78	0.0054	128	0.0156	1.806
rs12670064	154	158,869,415	C/T	0.1799	0.1627	0.1992	0.78	0.0045	119	0.0141	1.852
rs12670059	153	158,869,357	G/T	0.1796	0.1623	0.1989	0.78	0.0043	117	0.0137	1.864
rs7789969	150	158,865,207	C/T	0.1806	0.1632	0.2000	0.78	0.0043	115	0.0137	1.862
vr115 (rs55662704)	149	158,864,184	C/T	0.1046	0.0880	0.1230	0.69	5.31E-04	65	0.0030	2.522
vr114 (rs3793223)	146	158,863,574	A/C	0.1026	0.0862	0.1208	0.69	5.88E-04	72	0.0030	2.522

SNP	Sequential numbering (among 368 SNPs)	Position on Chr 7 (hg19)	Alleles minor/major	Minor allele frequency in			Odds ratio	Asymptotic P value	Rank	P_{cor}	$-\log_{10}(P_{cor})$
				All samples	Cases	Controls					
vr113 (rs3793222)	145	158,863,536	T/G	0.1009	0.0857	0.1178	0.70	0.0014	93	0.0055	2.258
rs3841524	143	158,862,493	A/AC	0.1684	0.1524	0.1862	0.79	0.0063	142	0.0163	1.788
vr112 (rs3815516)	140	158,862,177	A/T	0.1665	0.1524	0.1821	0.81	0.0173	186	0.0342	1.466
vr110 (rs1476998)	138	158,859,787	C/T	0.1745	0.1587	0.1921	0.79	0.0085	164	0.0190	1.722
vr109 (rs55864635)	137	158,859,744	T/C	0.1021	0.0854	0.1207	0.68	4.77E-04	62	0.0028	2.548
rs7782658	136	158,858,007	A/G	0.1727	0.1550	0.1925	0.77	0.0031	109	0.0104	1.983
vr108 (rs3793220)	135	158,857,436	A/G	0.1094	0.0989	0.1210	0.80	0.0223	194	0.0423	1.374
rs149678428	132	158,856,820	C/CG	0.1020	0.0892	0.1163	0.74	0.0050	126	0.0147	1.831
rs147875403	131	158,856,795	C/CAT	0.0776	0.0690	0.0871	0.78	0.0252	195	0.0475	1.324
rs116176153	130	158,855,902	G/A	0.1102	0.0931	0.1293	0.69	5.24E-04	63	0.0031	2.514
vr106 (rs114961653)	128	158,855,860	G/A	0.1099	0.0929	0.1289	0.69	4.70E-04	61	0.0028	2.547
7:158855518	127	158,855,518	A/AGAGAGCTGATTGGTCTGTTTTACT	0.1027	0.0868	0.1203	0.70	8.15E-04	83	0.0036	2.442
vr105 (rs7787641)	126	158,855,438	G/A	0.1032	0.0857	0.1226	0.67	3.22E-04	35	0.0034	2.470
vr104 (rs2540345)	125	158,855,420	C/T	0.1058	0.0893	0.1242	0.69	7.83E-04	81	0.0036	2.449
vr103 (rs56375711)	123	158,854,935	A/G	0.1009	0.0878	0.1154	0.74	0.0064	144	0.0163	1.787
rs2730267	122	158,854,873	C/T	0.1766	0.1595	0.1956	0.78	0.0049	123	0.0146	1.837
vr102 (rs3812302)	121	158,854,723	G/C	0.1672	0.1490	0.1876	0.76	0.0022	101	0.0081	2.090
rs73169224	118	158,850,818	T/C	0.0219	0.0158	0.0287	0.55	3.33E-04	40	0.0031	2.514
rs2467807	117	158,849,882	A/T	0.1920	0.1720	0.2142	0.76	0.0014	92	0.0055	2.263
vr101 (rs3793217)	116	158,848,821	G/A	0.1030	0.0877	0.1200	0.70	0.0015	97	0.0055	2.256
rs73169221	111	158,846,316	A/T	0.1206	0.1015	0.1419	0.68	2.08E-04	26	0.0029	2.532
vr099 (rs73169220)	110	158,846,197	C/A	0.1247	0.1041	0.1476	0.67	8.66E-05	20	0.0016	2.798
vr096 (rs56236179)	106	158,843,088	A/G	0.1273	0.1084	0.1483	0.70	3.34E-04	41	0.0030	2.523
vr095 (rs2540357)	104	158,842,375	G/C	0.1894	0.1695	0.2116	0.76	0.0015	96	0.0056	2.254
rs60691503	100	158,839,261	T/TA	0.1284	0.1079	0.1511	0.68	1.62E-04	23	0.0026	2.586
7:158838584	99	158,838,584	A/AT	0.0114	0.0076	0.0156	0.48	0.0089	165	0.0198	1.704
rs56697795	98	158,838,583	A/AT	0.0114	0.0076	0.0156	0.48	0.0089	166	0.0197	1.706
7:158838582	97	158,838,582	A/ATATATATAT	0.0114	0.0076	0.0156	0.48	0.0089	167	0.0196	1.709
7:158838578	96	158,838,578	T/TATAATATATA	0.0117	0.0078	0.0160	0.48	0.0077	161	0.0177	1.752
rs10274787	93	158,837,558	G/A	0.1259	0.1058	0.1483	0.68	1.76E-04	24	0.0027	2.568
rs2730252	92	158,837,453	C/T	0.0121	0.0084	0.0163	0.51	0.0169	185	0.0336	1.474
vr094 (rs73169218)	91	158,836,906	A/T	0.1225	0.1041	0.1430	0.70	4.04E-04	49	0.0030	2.518
vr093 (rs7784586)	90	158,836,379	C/T	0.1243	0.1052	0.1456	0.69	2.82E-04	29	0.0036	2.447
vr092 (rs2540359)	89	158,836,285	G/A	0.1252	0.1063	0.1463	0.69	3.28E-04	36	0.0034	2.475
vr091 (rs2730254)	88	158,836,247	C/G	0.1259	0.1073	0.1466	0.70	4.25E-04	52	0.0030	2.521
rs56294245	87	158,836,074	T/TGA	0.1187	0.1014	0.1378	0.71	7.59E-04	80	0.0035	2.457
rs2540353	86	158,835,631	A/G	0.0139	0.0099	0.0183	0.54	0.0212	193	0.0405	1.393
vr090 (rs73169216)	85	158,834,662	A/G	0.1220	0.1036	0.1424	0.70	4.03E-04	48	0.0031	2.510
vr089 (rs73169215)	84	158,833,701	C/T	0.1223	0.1036	0.1430	0.69	3.31E-04	39	0.0031	2.505
vr088 (rs886656)	83	158,832,638	A/G	0.1234	0.1057	0.1431	0.71	6.89E-04	76	0.0033	2.477
rs147666851	82	158,832,289	T/TGGGTGGGGCCTTGCCACAG	0.1258	0.1076	0.1462	0.70	5.32E-04	66	0.0030	2.527
rs1808215	81	158,831,888	A/G	0.1216	0.1034	0.1417	0.70	4.49E-04	55	0.0030	2.523
vr087 (rs2730224)	80	158,831,706	A/G	0.1272	0.1097	0.1466	0.72	0.0010	88	0.0041	2.382
rs2527185	79	158,829,966	G/A	0.0103	0.0069	0.0141	0.49	0.0100	170	0.0217	1.663
vr078 (rs2730220)	71	158,822,058	T/C	0.0936	0.0801	0.1086	0.72	0.0010	87	0.0040	2.394
vr067 (rs115079694)	59	158,818,622	T/C	0.0848	0.0757	0.0949	0.78	0.0203	192	0.0389	1.410
vr004 (rs262136)	1	158,764,915	T/C	0.0935	0.0809	0.1075	0.73	0.0017	100	0.0064	2.194

* For each group of variants, the row containing the variant giving the lowest asymptotic P value is highlighted in pink.
For each group of variants, the rows are highlighted in grey if the variants are in weak LD with the remaining variants of the group
"Rank" refers to the sequential order of the asymptotic P values (association test by SNPTEST) from the smallest to the largest with the smallest P value having a rank of 1.
"P_{cor}" refers to the P value corrected for multiple testing using the Benjamini-Hochberg procedure at a false discovery rate of 0.05

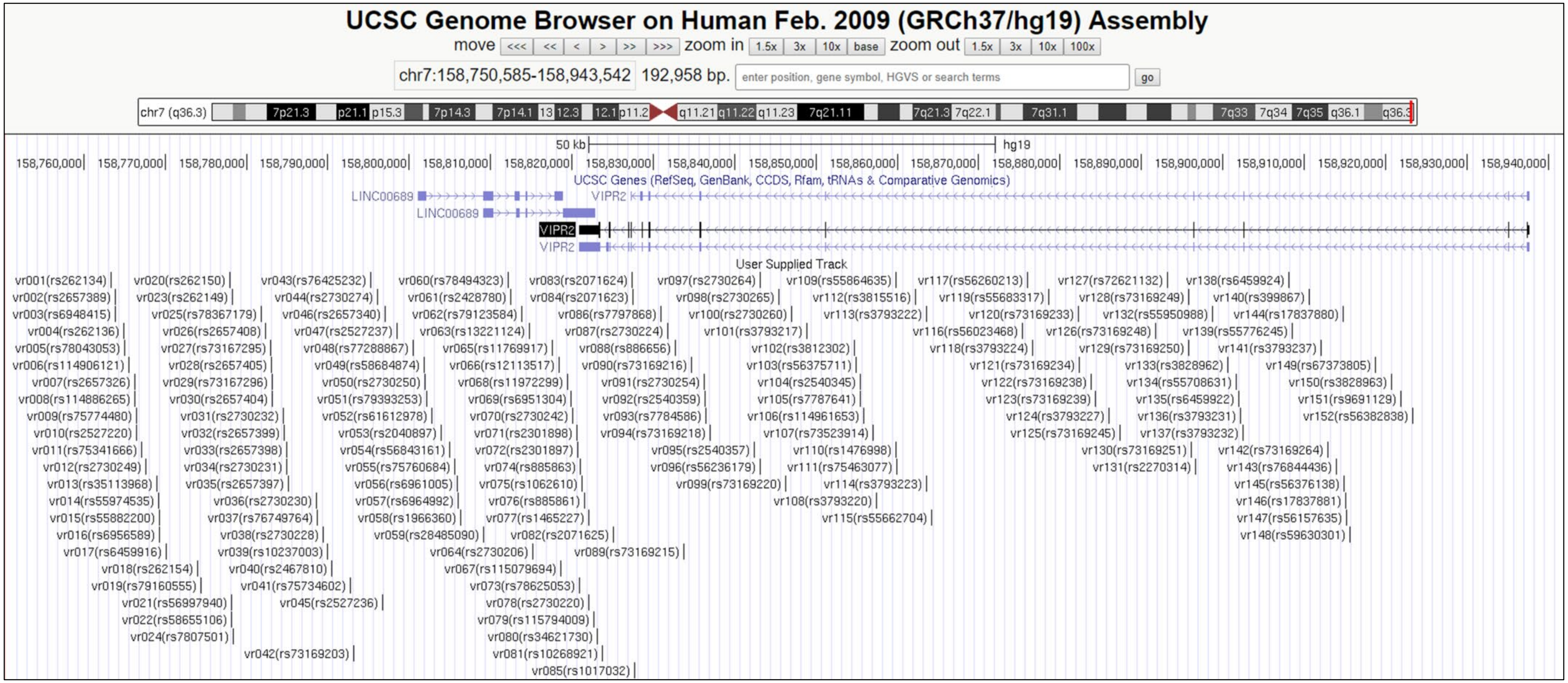

Supplementary Figure 1. The locations of single-nucleotide polymorphisms (SNPs) tested in Stage 1 as arranged along the plus strand of chromosome 9. The SNPs are coded as vr001 to vr152 in this article for the sake of convenience along with their respective rsid numbers sequentially along the chromosomal positions (GRC37/hg19). Note that the sense of *VIPR2* is on the minus strand of chromosome 7.

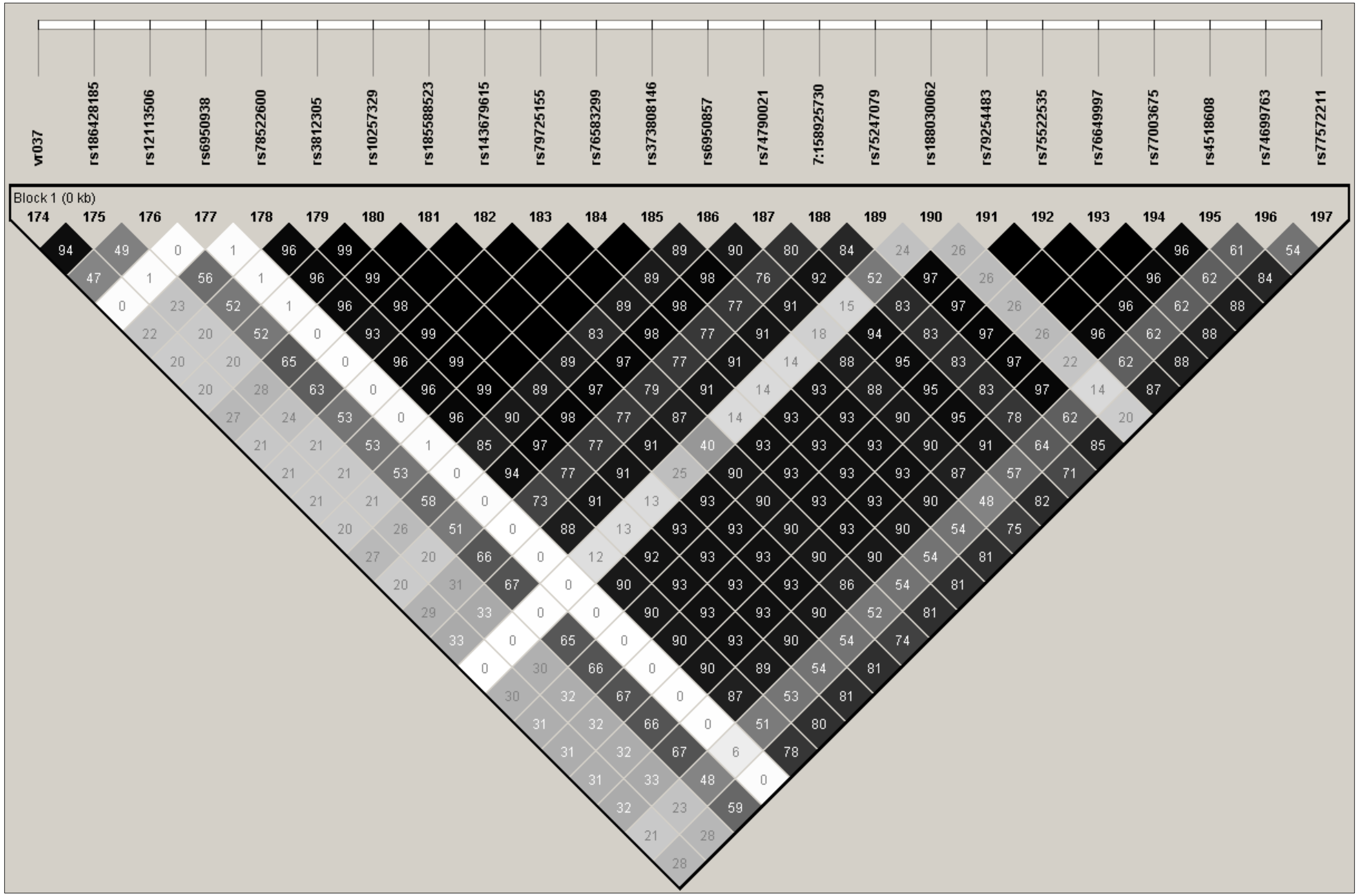

Supplementary Figure 2. The linkage disequilibrium (LD) pattern of high-risk variants identified in Stage-4 study. The LD measures are shown as r^2 values for cases and controls together for 24 variants with high-risk minor alleles. The high-risk variants are arranged from left to right sequentially according to their chromosomal positions on the plus strand of chromosome 7.

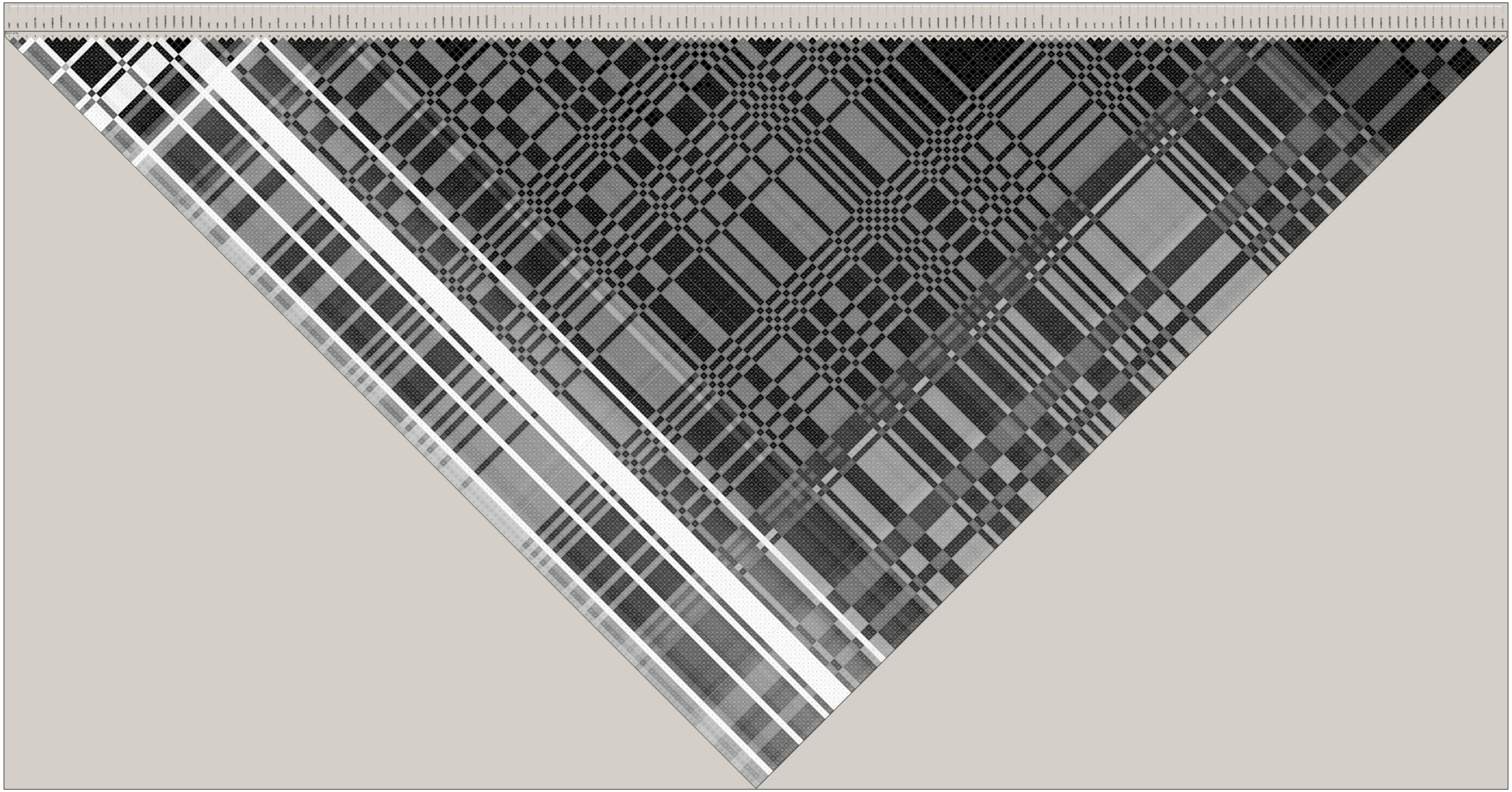

Supplementary Figure 3. The linkage disequilibrium (LD) pattern of protective variants identified in Stage-4 study. The LD measures are shown as r^2 values for cases and controls together for 173 variants with protective minor alleles. The colour scheme is as follows: black indicating $r^2=1$, white indicating $r^2=0$, and shades of grey indicting $0<r^2<1$. The protective variants are arranged from left to right sequentially according to their chromosomal positions on the plus strand of chromosome 7.

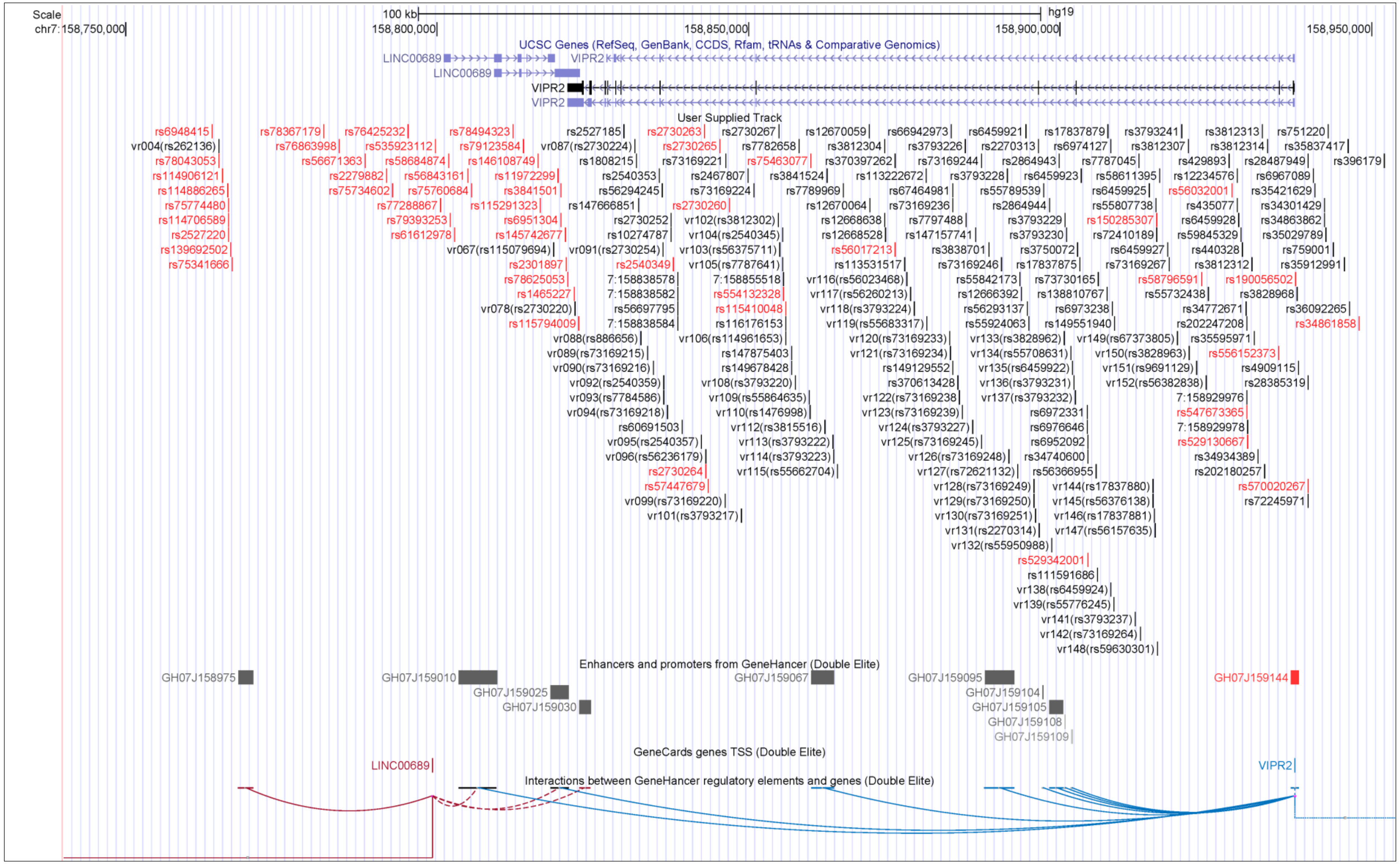

Supplementary Figure 4. The locations of protective variants, promoter and enhancers and the gene-enhancer interactions in the *VIPR2* region. Protective variants (indicated in black) and other variants in strong LD with them ($r^2 \geq 0.7$, indicated in red) are shown together with regulatory genomic elements (promoter and enhancers). Gene-enhancer interactions are indicated by arcs linking relevant regulatory elements. Note that some variants are located in the promoter or enhancers.

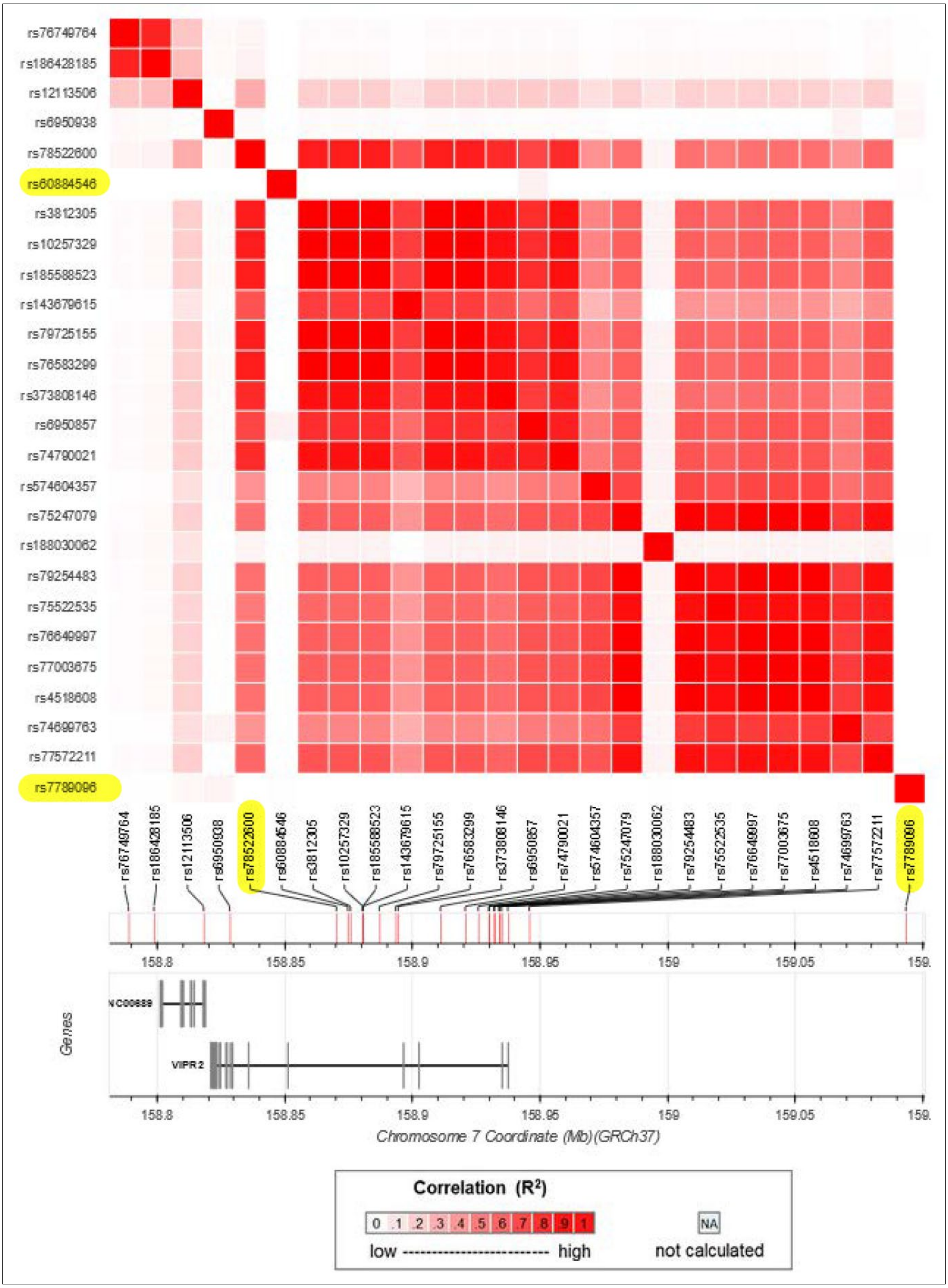

Supplementary Figure 5. The linkage disequilibrium (LD) pattern of high-risk variants identified in Stage-4 study and the "high-risk" variants (rs60884546 and rs7789096; highlighted in yellow) identified in a recent mega-study of refractive errors. The LD measures are shown as r^2 values for Chinese (CHB) based on the 1000 Genomes Database. The graph was produced using LDmatrix (https://ldlink.nci.nih.gov/?tab=ldmatrix).
